# Dual-template engineering of triple-layered nanoarray electrode of metal chalcogenides sandwiched with hydrogen-substituted graphdiyne

**DOI:** 10.1038/s41467-018-05474-0

**Published:** 2018-08-07

**Authors:** Sifei Zhuo, Yusuf Shi, Lingmei Liu, Renyuan Li, Le Shi, Dalaver H. Anjum, Yu Han, Peng Wang

**Affiliations:** 1 Water Desalination and Reuse Center, Biological and Environmental Science and Engineering Division, King Abdullah University of Science and Technology, Thuwal, 23955-6900 Saudi Arabia; 2 Advanced Membranes and Porous Materials Center, Physical Sciences and Engineering Division, King Abdullah University of Science and Technology, Thuwal, 23955-6900 Saudi Arabia; 3 Imaging and Characterization Core Lab, King Abdullah University of Science and Technology, Thuwal, 23955-6900 Saudi Arabia

## Abstract

Hybrid nanostructures integrating electroactive materials with functional species, such as metal-organic frameworks, covalent organic frameworks, graphdiyne etc., are of significance for both fundamental research and energy conversion/storage applications. Here, hierarchical triple-layered nanotube arrays, which consist of hydrogen-substituted graphdiyne frameworks seamlessly sandwiched between an outer layer of nickel–cobalt co-doped molybdenum disulfide nanosheets and an inner layer of mixed cobalt sulfide and nickel sulfide (Co_9_S_8_/Ni_3_S_2_), are directly fabricated on conductive carbon paper. The elaborate triple-layered structure emerges as a useful hybrid electrode for energy conversion and storage, in which the organic hydrogen-substituted graphdiyne middle layer, with an extended π-conjugated system between the electroactive nanomaterials, provides built-in electron and ion channels that are crucial for performance enhancement. This dual-template synthetic method, which makes use of microporous organic networks to confine a self-template, is shown to be versatile and thus provides a promising platform for advanced nanostructure-engineering of hierarchical multi-layered nanostructures towards a wide range of electrochemical applications.

## Abstract (web summary)

Multi-shelled nanomaterials offer interesting electrochemical properties, but have been limited in composition. Here the authors use dual templating to integrate electroactive metal chalcogenide layers with hydrogen-substituted graphdiyne, achieving electrocatalytic activity for hydrogen evolution.

## Introduction

Hollow nanomaterials with functional shells and inner voids are promising for advanced energy conversion and storage applications^1–5^. Recently, a variety of multi-layered hollow nanomaterials of metal-organic frameworks (MOFs)^6^, carbon nanospheres^7^, organosilica^8^, metal oxides^9^, sulfides^10,11^, and phosphides^12^ have been shown to outperform the conventional fully solid and single-shelled counterparts and show potential for contributing to the sustainable development of society. For example, multi-shelled hollow nanospheres^13,14^, nanopolyhedra^15,16^, and nanotubes^17^ are able to deliver enhanced electrochemical activities in Li-ion batteries and water splitting compared with their single-shelled counterparts. However, up to now, the multi-shells of these hollow nanomaterials are restricted to the same chemical composition^6–8,10–14,17^ or reduplicative geometric morphology of yolk-shell structure^12–16^, which limits their potential applications. Recently, integration of electroactive nanomaterials with functional species such as MOFs^18,19^, covalent organic frameworks (COFs)^20^, complexes^21^, graphdiyne^22^, and carbon^23–30^ etc., have experienced rapid development and have been widely applied in electrochemical-based catalysis and energy storage. In typical electrochemical applications, well-aligned nanoarrays directly grown on current collectors without any polymeric binder are considered as favorable electrode materials^31–34^. Furthermore, hybrid nanoarrays with multi-compositions show enhanced performance owing to synergistic functions^31,35–37^. Although various hollow hybrid nanoarrays have been synthesized, they are mainly single- and double-shelled nanostructures with all-inorganic components^33,35,37^. So far, the synthesis of hierarchical triple-layered hollow hybrid nanoarrays that have different layer geometric morphologies and chemical compositions and that can be directly used as electrode material, although attractive, does not exist at the moment to the best of our knowledge.

Self-templating approaches, in which sacrificial templates are involved, are widely used in controllable fabrication of complex hollow nanostructures due to the availability of various sacrificial templates^31,34,38–43^. However, mainly due to the paucity of suitable self-templates, this approach has not been successful in synthesizing triple-layered hollow nanostructures in which each shell has different compositions and morphologies. On the other hand, diverse microporous organic networks (MONs) with great chemical and thermal stability have recently been prepared through various chemical coupling reactions^44–49^. Cross-linked carbon-rich frameworks of MONs, such as hydrogen-substituted graphydiyne (HsGDY) with a highly extended π-conjugated system, have shown satisfactory conductivity and ion diffusion, leading to application in flexible electrodes for Li/Na storage^50^. In addition, the applications of HsGDY have been gradually extended to organic catalysis^51,52^ and photocatalysis^53^. Recently, it has been reported that HsGDY is able to form a rigid conformal microporous coating layer on the surface of many self-templating materials due to the robustness and well-defined porosity of HsGDY^51^. The HsGDY conformal coating layer is expected to have selective species permeability. Therefore, a self-template with a conformally coated HsGDY layer would permit the migration of species from the inner self-template region through the HsGDY and into the external surface of the HsGDY layer where they can be designed to react with desirable species in bulk solution and vice versa. Therefore, it is expected that an HsGDY-coated self-template is an interesting platform to synthesize triple-layered nanostructures. In this case, both the HsGDY coating layer and the inner confined self-template serve the role of template and can be considered as dual-template. The dual-template strategy that involves a HsGDY-coated self-template has the potential for producing triple-layered nanoarrays with specific chemical compositions and morphologies for each layer.

Using electricity that is generated by a photovoltaic cell as an energy source for electrocatalytic water splitting is considered to be a clean-energy technique for hydrogen production. In pursuit of a cost-effective high-performance electrocatalyst for the hydrogen evolution reaction (HER), new nanomaterial design concepts and strategies have attracted considerable efforts^54,55^. Molybdenum-based ternary sulfides M-MoS_x_ (M = Ni, Co, Fe, Zn, Cu) have been identified as promising electrocatalysts for water splitting under a wide range of pH values due to a synergistic effect between M and Mo^56–61^. A number of theoretical and experimental studies indicate that doping heteroatoms into the MoS_2_ matrix stimulates the intrinsic activity of in-plane S atoms^56,57,60,61^. Recently, integration of MoS_2_ with other functional materials, such as carbon^62^, reduced graphene oxide (r-GO)^63^, metal complexes^21^, and other transition metal sulfides^36,64,65^, has led to enhanced HER performance. The availability of the various Ni- and Co-based nanostructures makes them promising candidates for the construction of hierarchical nanostructures, along with MoS_2_, for enhanced electrocatalytic performance. Thus, the integration of multiple electroactive metal chalcogenides with HsGDY, especially within a multi-layered nano-framework directly on a conductive substrate, is highly desirable for HER-based applications.

**Fig. 1:**
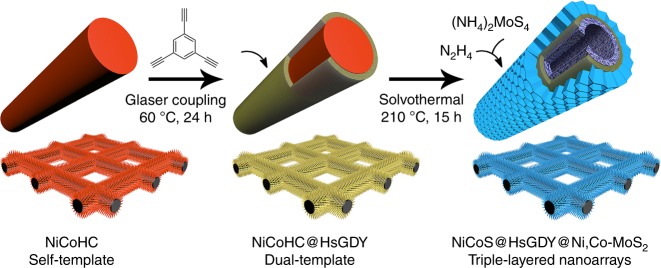
Schematic illustration of the fabrication of triple-layered nanotube arrays. The chemical synthesis of hierarchical triple-layered NiCoS@HsGDY@Ni,Co-MoS_2_ nanoarray comprised of Ni_3_S_2_/Co_9_S_8_ inner layer (NiCoS), hydrogen-substituted graphdiyne (HsGDY), and Ni-,Co-co-doped MoS_2_ (Ni,Co-MoS_2_) is achieved with a HsGDY-coated (Ni,Co)(OH)_2_CO_3_ (NiCoHC) nanoarray as a dual template

Here, we provide a proof-of-concept of using an HsGDY-coated self-template as a dual template to fabricate triple-layered nanostructures. We demonstrate the synthesis of hierarchical triple-layered nanoarrays of transition metal chalcogenides (TMCs) directly on a carbon paper by using HsGDY-coated nickel cobalt hydroxyl carbonate (NiCoHC) nanowire arrays as a dual template. The synthesized material has built-in HsGDY nanolayers sandwiched between Ni-, Co-co-doped MoS_2_ (Ni,Co-MoS_2_) nanosheets, and mixed NiCoS (Co_9_S_8_, Ni_3_S_2_) nanotubes. The synthesis involves preparation of NiCoHC nanowire arrays as a bimetallic self-template, confinement of the NiCoHC nanowire arrays with conformally coated HsGDY layers (NiCoHC@HsGDY), and transformation of NiCoHC@HsGDY into tri-layered NiCoS@HsGDY@Ni,Co-MoS_2_ (Fig. 1). The microporous HsGDY layer (step II) in NiCoHC@HsGDY acts as not only a physical separator to confine the in-situ conversion of NiCoHC nanowires to mixed NiCoS nanotubes in the interior, but also a chemical nucleation platform with an ion channel to generate Ni,Co-MoS_2_ nanosheets on the external surface (step III). This triple-layered nanoarray on carbon paper incorporates inorganics (i.e., TMCs) with organics (i.e., HsGDY), and the intermediate HsGDY layer can work as a built-in electron and ion channel owing to the highly extended π-conjugated system, all pointing toward a potential for being used directly as a binder-free and self-supported electrode. Due to the hierarchical nanostructures, the enhanced number of active sites, improved electron conductivity and mass transport of the geometric constraint, the material shows high current density, low overpotential, and excellent anti-aggregation behaviors over a wide pH range (0–14) in HER. The dual-template approach is further extended to prepare other triple-layered hollow nanostructures of transition metals, such as Ni_3_S_2_@HsGDY@Ni-MoS_2_ nanosheet arrays and Co_9_S_8_@HsGDY@Co-MoS_2_ nanotube arrays, proving its versatility.

## Results

### Construction of the dual-template

**Fig. 2:**
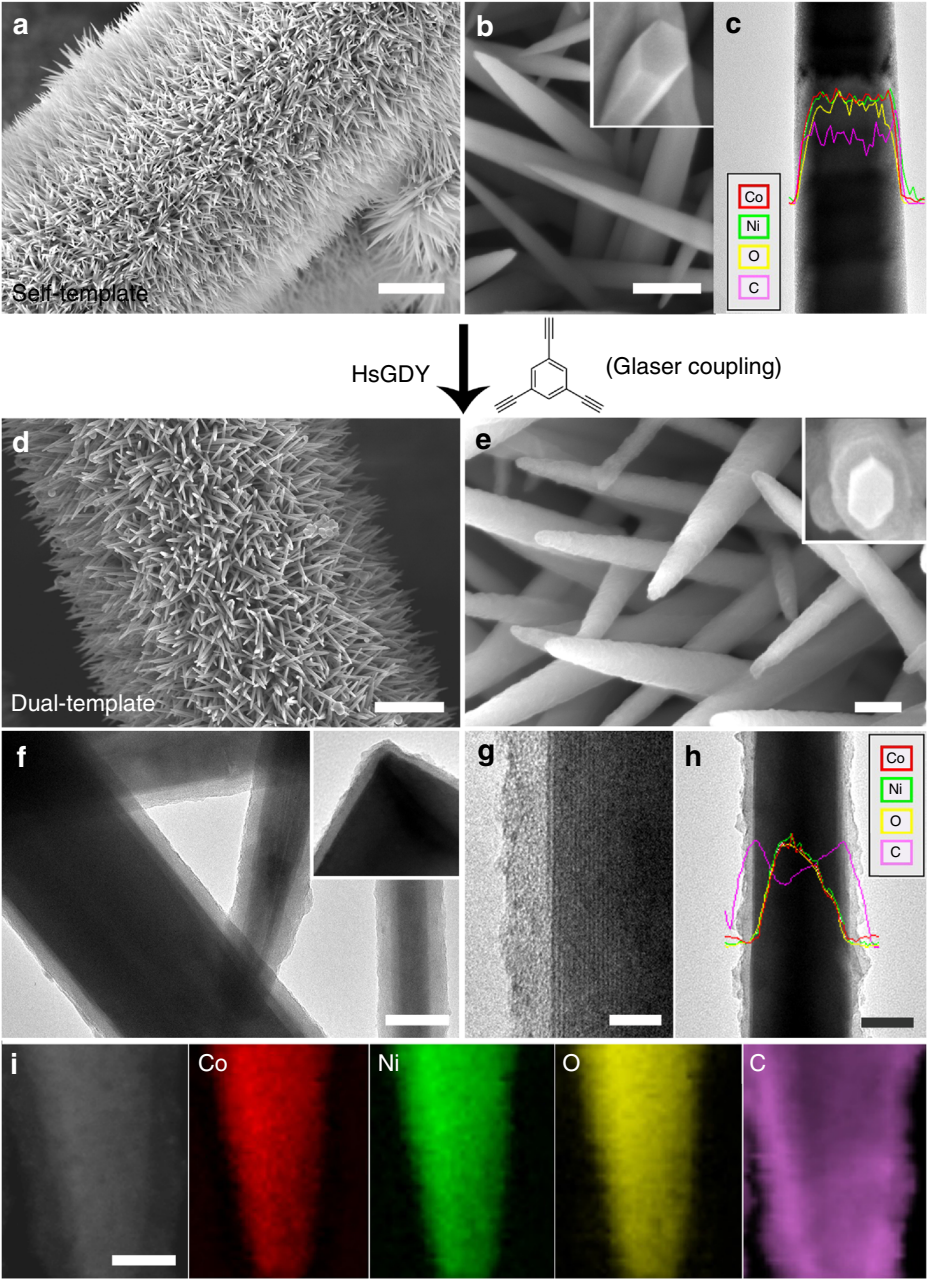
Scanning electron microscopy and transmission electron microscopy micrographs. **a**, **b** Scanning electron microscopy (SEM) images, **c** Transmission electron microscopy (TEM) image and line-scan electron energy loss spectroscopy (EELS) elemental distributions of the (Ni,Co)(OH)_2_CO_3_ (NiCoHC) nanowires arrays. **d**, **e** SEM images, **f**, **g** TEM images, **h** line-scan EELS elemental distributions, and **i** Scanning transmission electron microscopy (STEM)-EELS elemental mapping of the NiCoHC@hydrogen-substituted graphdiyne (HsGDY) nanowires. The insets of **b**, **e**, and **f** are the corresponding SEM images of the cross-sections and TEM image of the corner, respectively, taking from some broken nanofibres. Scale bars: (**a**, **d**) 2 μm, (**b**, **e**) 200 nm, (**f**, **h**, **i**) 50 nm, (**g**) 10 nm

In this work, the conformally coated microporous HsGDY layers are the key to the dual-template approach. It serves not only as a conformal separator to confine the self-template, but also acts as a mass transport channel and rigid scaffold for the nucleation and growth of the third layer on the external surfaces. In detail, well-aligned NiCoHC nanowire arrays are first prepared directly on carbon fibre paper in virtue of the bimetallic character and availability of the various Ni- and Co-based nanostructures^32^. As shown in the scanning electron microscopy (SEM) images (Fig. 2a, b and Supplementary Fig. 1), well-aligned tapering nanowire arrays with diameters of ~100–200 nm and lengths up to 2 μm are uniformly distributed on the carbon paper. The radial growth of these nanowires directly around the carbon fibres and the tapering character of each nanowire leads to increased surface area and thus are potentially beneficial for many applications. The unimodal profile and uniform spatial distribution of elemental Ni, Co, O, and C, as shown in the line-scan electron energy loss spectroscopy (EELS) profiles (Fig. 2c) and scanning transmission electron microscopy-electron energy loss spectroscopy (STEM-EELS) elemental mapping (Supplementary Fig. 2d), respectively, indicate the solid nature of these nanowires with bimetallic characteristics. The characteristic vibration and diffraction peaks shown in the Fourier transform infrared (FTIR) spectra (Supplementary Fig. 7) and X-ray diffraction (XRD) patterns (Supplementary Fig. 3), respectively, further identify these nanoarrays as hydroxyl-carbonate, (Ni,Co)(OH)_2_CO_3_, with high crystallinity^32,37^. Inductively coupled plasma-atomic emission spectrometry (ICP-AES) reveals the atomic ratio of Co/Ni as about 2, which indicates the composition of NiCoHC as Ni_0.67_Co_1.33_(OH)_2_CO_3_. These results demonstrate that bimetallic NiCoHC nanowire arrays have been successfully fabricated as the self-template.

Then, a layer of HsGDY is directly coated onto each NiCoHC nanowire to form a coating by Glaser coupling of 1,3,5-triethynylbenzene^51^. As shown in the SEM (Fig. 2d, e and Supplementary Fig. 4) and transmission electron microscopy (TEM) images (Fig. 2f and Supplementary Fig. 5a–c), the HsGDY on each nanowire inherits well-aligned and tapering character of the underlying self-template of the NiCoHC nanowire, indicating the seamless conformal coating of HsGDY. As shown in Fig. 2f, a thin and uniform layer perfectly covers and preserves the underlying morphology of NiCoHC at the right corner. The thickness of the HsGDY coating is around 10 nm. The straight boundaries between the crystalline NiCoHC nanowires and HsGDY layers are clearly distinguishable and further confirm the conformal coating nature of the HsGDY layers (Fig. 2g). The XRD patterns of NiCoHC do not show any changes before and after HSGDY coating (Supplementary Fig. 3), which is consistent with TEM observations. Obviously, the corresponding elemental distribution of C, with wider coverage as compared with Ni, Co, and O (Fig. 2h, i and Supplementary Fig 5e), supports the full and uniform coating of NiCoHC nanowires by the HsGDY.

**Fig. 3:**
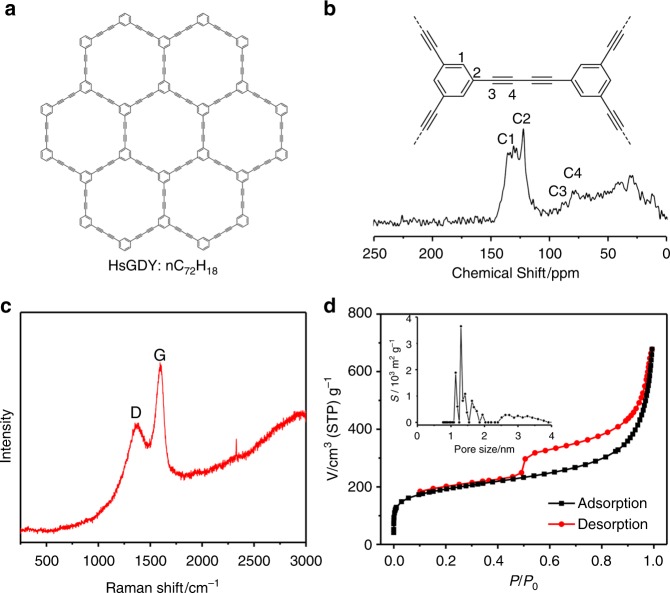
Structural characterizations of hydrogen-substituted graphdiyne. **a** Schematic illustration, **b** solid ^13^C nuclear magnetic resonance (NMR) spectrum, **c** Raman spectrum, and **d** N_2_ adsorption/desorption isotherms of the discerte conformal coated hydrogen-substituted graphdiyne (HsGDY). The inset of **d** is the corresponding pore size distribution diagram

To ascertain the chemical composition and porosity of the HsGDY coatings, some discrete HsGDY nanotubes are intentionally fabricated by removing the NiCoHC by acid etching process, which would otherwise affect the ^13^C nuclear magnetic resonance (NMR) analysis due to the presence of paramagnetic species (Co^2+^) and interfere with the N_2_ adsorption/desorption (Supplementary Fig. 6). The isolated HsGDY sample is characterized using NMR, X-ray photoelectron spectroscopy (XPS), Raman, and FTIR spectra. The solid-phase ^13^C NMR spectrum (Fig. 3b) shows the signals at *δ* = 136.2 ppm and 123.1 ppm assigned to the *sp*^2^-hybridized aryl and peaks at *δ* = 90.3 ppm and 81.5 ppm, which can be ascribed to the *sp*-hybridized alkyne, and thus indicates the highly conjugated electronic structure of the as-prepared HsGDY layers with extended π-conjugation system bearing *sp*- and *sp*^2^-hybridized carbon atoms^50,51,66^. This result is in accordance with the XPS results (vide infra) with sub-peaks at 284.3 and 285 eV attributed to the C 1 *s* orbital of C=C (*sp*^2^) and C≡C (*sp*), respectively^50,67^. Besides, the peaks of 1370 cm^−1^ and 1597 cm^−1^ of the Raman spectrum correspond to the D band and G band of vibration of *sp*^2^ carbon domains in aromatic rings, respectively (Fig. 3c)^67^, and the weak peaks located at 2327 cm^−1^ can be assigned to the alkyne-related stretching vibrations, revealing the large π-conjugation system of HsGDY cross-links aromatic rings with acetylenic bonds^50^. Furthermore, the representative vibration peaks for C≡C bond (2190 cm^−1^) and aromatic C-H (881 cm^−1^, 3060 cm^−1^) in the FTIR spectrum (Supplementary Fig. 7) identify the structure of HsGDY with *sp*- and *sp*^2^-hybridized carbon atoms as poly(phenylene butadiynylene)^50,52^. In addition, the analysis of the N_2_ isotherms (Fig. 3d) based on the Brunauer–Emmett–Teller (BET) theory indicates that the HsGDY layers possess micropores with diameters around 1.2 nm and a surface area up to 651 m^2^ g^−1^. Therefore, core-shell nanowire arrays comprised of a NiCoHC nanowires core and a conformal surface coating of rigid and microporous HsGDY as the shell are successfully achieved. The conformal and uniform coating of HsGDY on each self-template nanowire with coaxial character enable its use as a dual-template platform for the homogeneous growth of a third layer right on top of the HsGDY coating layer.

### Synthesis of the triple-layered nanotube arrays

**Fig. 4:**
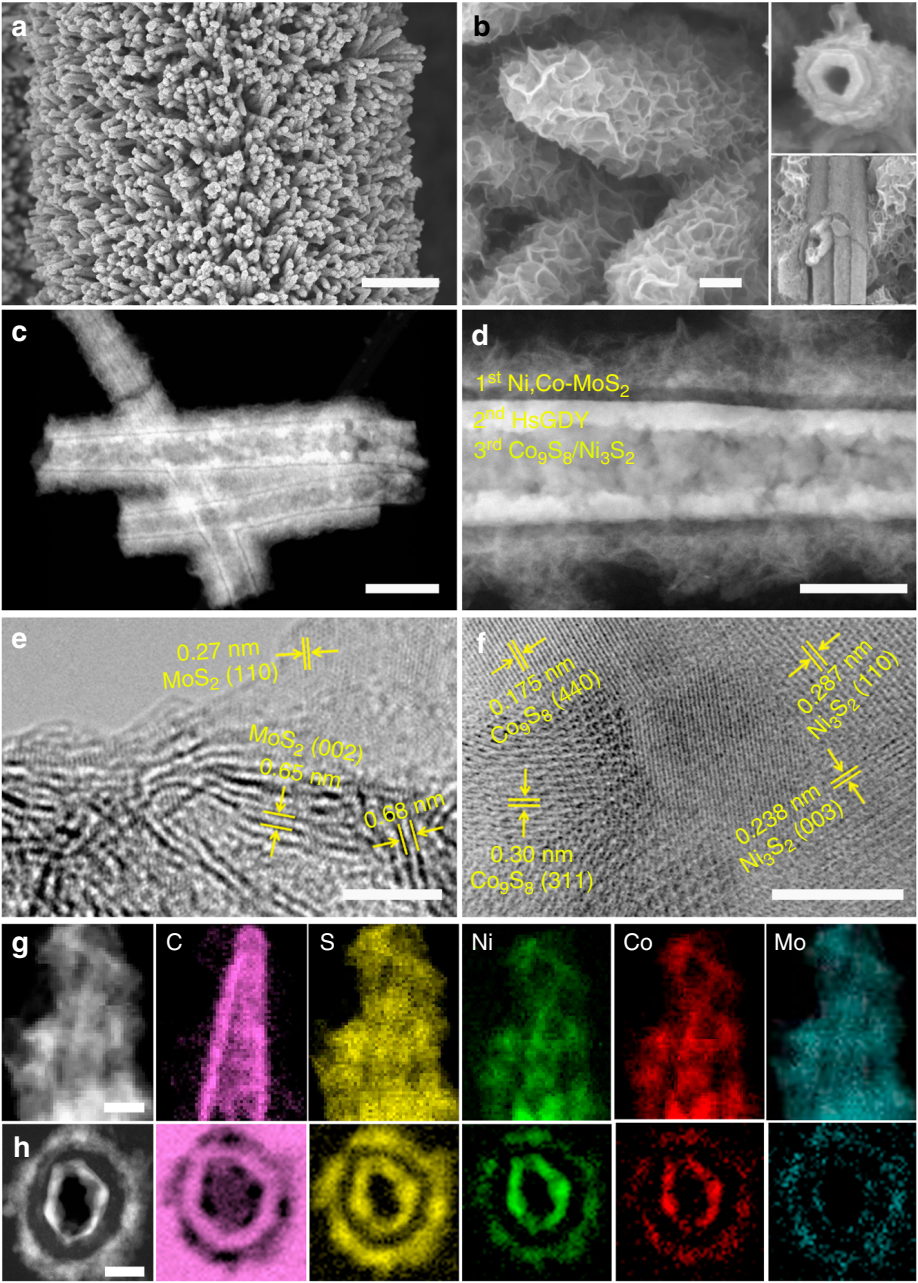
Micrographs of the tri-layered nanoarrays. **a**, **b** scanning electron microscopy (SEM) images, **c**, **d** high angle annular dark field-scanning transmission electron microscopy (HAADF-STEM) images, **e**, **f** high-resolution transmission electron microscopy (HRTEM) images, **g** side-view, and **h** microtome-prepared cross-section scanning transmission electron microscopy-energy loss spectroscopy (STEM-EELS) elemental mappings of the hierarchical triple-layered NiCoS@HsGDY@Ni,Co-MoS_2_ nanotubular arrays comprised of Ni_3_S_2_/Co_9_S_8_ inner layer (NiCoS), hydrogen-substituted graphdiyne (HsGDY), and Ni-,Co-co-doped MoS_2_ (Ni,Co-MoS_2_). The insets of **b** show some broken areas; **e** and **f** are collected at the external Ni,Co-MoS_2_ nanosheets and the interior NiCoS nanotubes, respectively. Scale bars: **a** 2 μm, **b**, **d** 100 nm, **c** 200 nm, **e**, **f** 5 nm, **g** 50 nm, **h** 20 nm

These as-prepared dual-template of NiCoHC@HsGDY are utilized to construct molybdenum-based ternary sulfides by reaction with NH_4_MoS_4_ in the presence of N_2_H_4_. The SEM images show, after the reaction, the well-aligned structure of the self-templating nanowires are preserved (Fig. 4a) with each nanowire fully covered with interconnected nanosheets (Fig. 4b and Supplementary Fig. 8). Close observation by TEM (Fig. 4c) and high magnification SEM (inset of Fig. 4b and Supplementary Fig. 8) at broken areas of the samples clearly reveal a triple-layered tube-in-tube nanostructure. High angle annular dark field-scanning transmission electron microscopy (HAADF-STEM) (Fig. 4d) confirms that the triple-layered structures are composed of an HsGDY middle layer seamlessly sandwiched between a flower-like and loosely assembled nanosheet outer layer and a hollowed-out nanotube inner layer. Impressively, the clear-cut and continuous layer boundaries on both sides of the HsGDY middle layer (Fig. 4d) suggest the firmly confined inner layer and the free growing outer layer. The exterior flower-like assembled nanosheets with well exposed edges and the interior hollowed-out nanotubes would greatly enhance the number of active sites in these triple-layer structures. High-resolution TEM (HRTEM) images and energy dispersive spectroscopy (EDS) are then collected to analyze elemental compositions of these three layers one by one. First, the outer layer of the nanosheets show an expanded interplanar spacing (e.g., 0.65 nm and 0.68 nm) and lattice distance of 0.27 nm (Fig. 4e and Supplementary Fig. 9), which are assigned solely to hexagonal MoS_2_ (2H-MoS_2_). The corresponding EDS spectrum reveals the presence of Ni and Co in the nanosheets (Supplementary Fig. 10), implying the incorporation of Ni and Co ions, thus insinuating outward diffusion of Ni and Co ions through HsGDY layers^57^. The uniform distribution of elemental Ni, Co, Mo, and S with encapsulated elemental C with distinguishable contrast in the side-view STEM-EELS elemental mapping (Fig. 4g) further identify the incorporation of Ni and Co ions within MoS_2_. Therefore, it is believed that the outer layer of the triple-layered structure is Ni-, Co-co-doped MoS_2_, denoted as Ni,Co-MoS_2_ hereafter. On the other hand, the hollowed-out nanotubes are identified as Co_9_S_8_ with lattice distance of 0.175 nm (440) and 0.30 nm (311), and Ni_3_S_2_ with lattice distance of 0.287 nm (110) and 0.238 nm (003) under HRTEM images (Fig. 4f and Supplementary Fig. 9)^68,69^. To further investigate the compositions of the inside layers, ultrathin sections perpendicular to the concentric shaft are prepared using ultramicrotome to cut the sample. As shown in Fig. 4h, the triple-layered structure is apparent by the onion-like structure of the cross-section. Notably, only C is detected in the middle layer, which suggests that no TMC residues block the micropores of the HsGDY layers. Furthermore, the elements of Ni, Co, and S distribute over both the inner and outer layers, while Mo dominantly accumulates around the outer layer, indicating that mixed NiCoS (Ni_3_S_2_, Co_9_S_8_) dominates the inner layer. The results so far support the successful fabrication of triple-layered nanoarrays. Efforts are then made to ascertain the detailed chemical compositions of each layer.

**Fig. 5:**
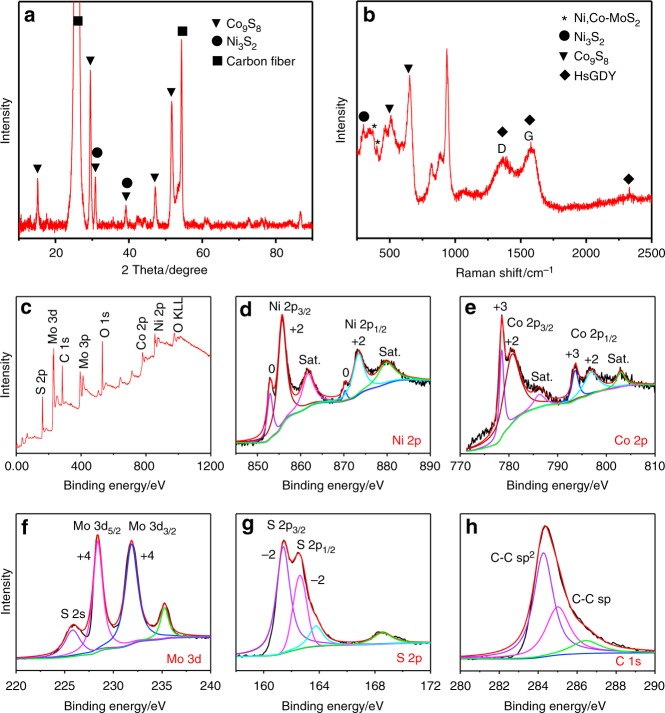
Component characterizations of the tri-layered nanoarrays. **a** X-ray diffraction (XRD) pattern, **b** Raman spectrum, **c** X-ray photoelectron spectroscopy (XPS) survey spectrum, and **d**–**h** high-resolution XPS spectra of Ni, Co, Mo, S, and C for the triple-layered NiCoS@HsGDY@Ni,Co-MoS_2_ nanotubes comprised of Ni_3_S_2_/Co_9_S_8_ inner layer (NiCoS), hydrogen-substituted graphdiyne (HsGDY), and Ni-,Co-co-doped MoS_2_ (Ni,Co-MoS_2_)

XRD, Raman, and XPS are further conducted to evaluate the detailed elemental compositions and chemical states of the triple-layered nanostructure. The well-resolved diffraction XRD peaks of cubic Co_9_S_8_ (JCPDS No.86-2273)^65^ are clearly seen, while the peaks of Ni_3_S_2_ and MoS_2_ are weak (Fig. 5a). However, Raman spectra (Fig. 5b and Supplementary Fig. 11) reveal the presence of Ni_3_S_2_ (297 cm^−1^ and 341 cm^−1^)^68^, Co_8_S_9_ (463 cm^−1^, 507 cm^−1^ and 651 cm^−1^)^69^, Ni,Co-MoS_2_, and HsGDY in the triple-layered nanostructure. The increased ID/IG value of HsGDY after the chemical transformation is attributed to the defects formed in the HsGDY during the solvothermal treatment process (Supplementary Fig. 12) and the peaks located at 817 cm^−1^, 882 cm^−1^, and 933 cm^−1^ can be assigned to Ni- and Mo-based oxides that are easily generated by laser irradiation during the Raman test^70^. Notably, the Raman bands at 379 cm^−1^ and 401 cm^−1^ correspond to the in-plane of E_2g_ and out-of-plane of A_1g_ modes of hexagonal MoS_2_, indicating the similar layered structure of Ni-,Co-co-doped MoS_2_ with MoS_2_^56^. The XPS survey spectrum confirms the presence of Ni, Co, Mo, S, and C with specific chemical states (Fig. 5c). In the high-resolution XPS spectrum of Ni 2p (Fig. 5d), the binding energies (BEs) at 855.7 eV and 873.4 eV with a spin energy of 17.7 eV are ascribed to Ni^2+^ 2*p*_3/2_ and Ni^2+^ 2*p*_1/2_, respectively, and the BEs of Ni^0^ 2*p* shown in 852.9 eV and 870.3 eV confirm the formation of Ni_3_S_2_^68,71^. The high-resolution XPS of Co 2*p* (Fig. 5e) can be deconvoluted into four primary peaks at 778.5 eV (Co^3+^), 780.8 eV (Co^2+^) for Co 2*p*_3/2_, and 793.6 eV (Co^3+^), 797.0 eV (Co^2+^) for Co 2*p*_1/2_, respectively, which could be assigned to Co_8_S_9_^69^. Specially, two sharp peaks located at 228.4 eV and 231.85 eV, attributed to Mo 3*d*_5/2_ and Mo 3*d*_3/2_, are also clearly observed, indicating the dominance of Mo^4+^ in the synthesized nanostructures (Fig. 5f)^57^. The presence of Mo^6+^ (3*d*_3/2_, 235.2 eV) in the XPS spectrum is attributed to the following: (1) the residual (NH_4_)_2_MoS_4_ species from the hydrothermal process can be adsorbed onto the surface of the tri-layered nanostructures^57^, and (2) partial surface oxidation in air can potentially lead to the presence of Mo^6+^ in these triple-layered nanostructures^72^. Fig. 5g shows the presence of typical coordination of sulfur with metal ions, located at 161.4 eV and 162.6 eV for S^2−^ 2*p*_3/2_ and S^2−^ 2*p*_1/2_, respectively. In addition, the C 1 *s* XPS spectrum (Fig. 5h) with two sub-peaks at 284.3 eV for C-C (*sp*^2^) and 285 eV for C-C (*sp*) verifies the existence of HsGDY and confirms that HsGDY is not changed during the chemical reaction^50^. These results verify that the synthesized triple-layered nanostructure has a chemical composition of NiCoS (Ni_3_S_2_, Co_9_S_8_)@HsGDY@Ni,Co-MoS_2_.

**Fig. 6:**
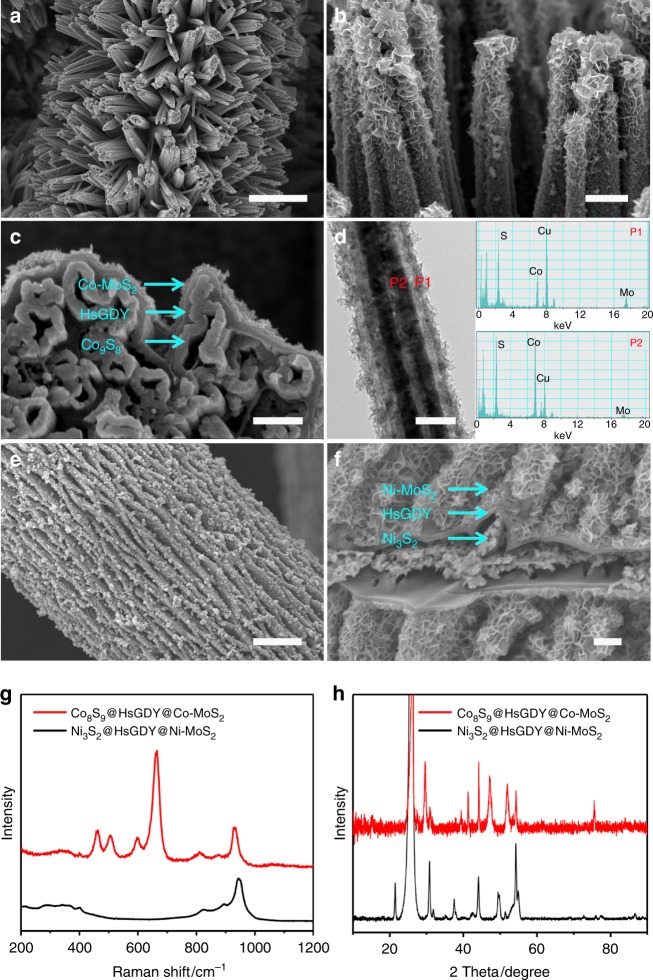
Structural characterizations of tri-layered nanoarrays. **a**–**c**, **e**, **f** Scanning electron microscopy (SEM) images, **d** transmission electron microscopy (TEM) image with corresponding energy dispersive spectroscopy (EDS) spectra, **g** Raman spectra, and **h** X-ray diffraction (XRD) patterns of the tri-layered Co_9_S_8_@ hydrogen-substituted graphdiyne (HsGDY)@Co-doped MoS_2_ (Co-MoS_2_) nanotube arrays (**a**–**d**) and Ni_3_S_2_@HsGDY@ Ni-doped MoS_2_ (Ni-MoS_2_) nanosheet arrays (**e**–**f**). Scale bars: **a** 5 μm, **b** 500 nm, **c**, **f** 200 nm, **d** 100 nm, **e** 2 μm

To better understand the mechanism of the transformation of NiCoHC@HsGDY nanowire arrays into the triple-layered NiCoS@HsGDY@Ni,Co-MoS_2_, additional control experiments are conducted. In the absence of N_2_H_4_, there are only Ni_x_Co_1-x_MoS_4_ nanoparticles formed on the HsGDY surfaces while the inner layer disappears (Supplementary Fig. 13g-i). In the absence of the HsGDY conformal coating layer, namely, in the presence of only the self-template of NiCoHC, a mixed single-layered NiCoMoS (Co_9_S_8_, Ni_3_S_2_, Ni,Co-MoS_2_) nanotubes are obtained, which is convincing evidence of the critical role of the HsGDY conformal coating layer in the successful synthesis of the triple-layered nanostructure (Supplementary Fig. 13d–f). Thus, we believe that the conformal coating of HsGDY layer serves two roles: (1) physical separation with ion channels to permit two different reaction spaces on its two sides, and (2) physical embrace that keeps the inner layer firmly in place. On the one hand, S^2−^ generated by the decomposition of (NH_4_)_2_MoS_4_ in the presence of N_2_H_4_ firstly diffuses inward across HsGDY to generate interior Ni_3_S_2_ and Co_8_S_9_. On the other hand, some Ni^2+^ and Co^2+^ originally in the self-template of NiCoHC are able to diffuse outward across the HsGDY to form Ni,Co-MoS_2_ on the external surface of HsGDY where they react with MoS_4_^2−^ in the presence of N_2_H_4_. In this work, further efforts are made to prove its versatility by synthesizing hollow triple-layered Co_9_S_8_@HsGDY@Co-MoS_2_ nanotube arrays (Fig. 6a–d) and Ni_3_S_2_@HsGDY@Ni-MoS_2_ nanosheet arrays (Fig. 6e, f, Supplementary Fig. 14) using the dual-template of cobalt hydroxyl-fluoride@HsGDY (Co(OH)F@HsGDY) and nickle hydroxyl-fluoride (Ni(OH)F@HsGDY), respectively. Both of the dual templates are grown directly on carbon paper and the details of these synthesis can be found in Supplementary Methods 1.

### Electrochemical performance as electrodes

**Fig. 7:**
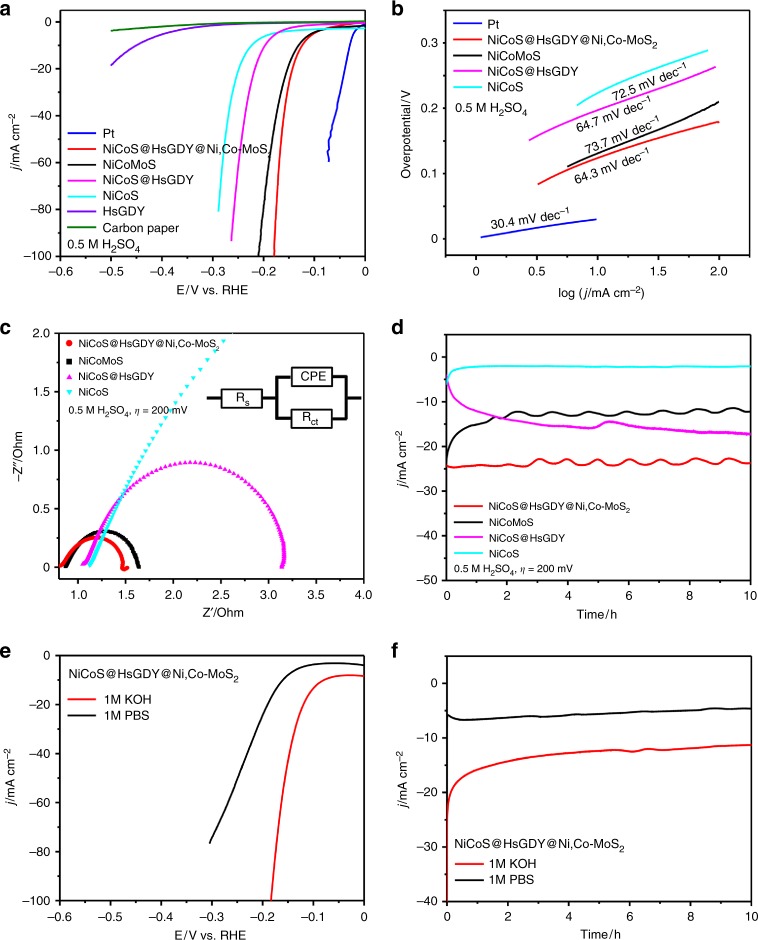
Electrochemical evaluation of hydrogen evolution reaction performance. **a** Hydrogen evolution reaction (HER) polarization curves with *iR*-compensations, **b** Tafel plots, **c** Nyquist plots derived from electrochemical impedance spectroscopy (EIS), with an equivalent circuit (CPE constant phase element, *R*_s _equivalent series resistance, *R*_ct _charge transfer resistance), **d** time-dependent current density curves without *iR*-compensations of Ni_3_S_2_/Co_9_S_8_ (NiCoS)@hydrogen-substituted graphdiyne (HsGDY)@Ni-,Co-co-doped MoS_2_ (Ni,Co-MoS_2_), mixed Co_9_S_8_, Ni_3_S_2_, and Ni,Co-MoS_2_ (NiCoMoS), NiCoS@HsGDY, NiCoS, HsGDY, Pt sheet and Carbon paper in 0.5 M H_2_SO_4_ (pH = 0.5). **e** HER polarization curves with *iR*-compensations and **f** time-dependent current density curves without *iR*-compensations of NiCoS@HsGDY@Ni,Co-MoS_2_ in 1 M KOH (pH = 14.1) and 1 M phosphate buffer solution (PBS) (pH = 7.1), respectively

Based on the conductivity and ion diffusion capability of the HsGDY middle layer in the synthesized triple-layered nanostructure and the fact that the triple-layered nanostructures are grown on conductive carbon paper, the performance of the material as an electrode for HER over a wide range of pH 0–14 is assessed. To substantiate the advantage of the tri-layered nanostructure in this work and to reveal the function of the HsGDY in the nanotube arrays (Supplementary Fig. 15), NiCoS, NiCoS@HsGDY, and mixed NiCoMoS (Ni_3_S_2_, Co_9_S_8_, Ni,Co-MoS_2_) (Supplementary Fig. 16) are further fabricated purposefully as contrast electrodes. To directly reflect the intrinsic electrocatalytic behavior of these tri-layered nanoarrays, an (*iR*) correction was applied to compensate the ohmic potential drop lossing from the solution resistance, i.e., the overpotential presented in Fig. 7a was corrected by *iR*, where *i* means the current and *R* refers to the Ohmic electrolyte resistance acquired by impedance measurements. As shown in Fig. 7a, the increase in HER performance from NiCoS, to NiCoS@HsGDY and further to NiCoS@HsGDY@Ni,CoMoS_2_ indicates the role of each layer and the necessity of the tri-layered structure. More specifically, NiCoS@HsGDY@Ni,Co-MoS_2_ delivers an overpotential of 124 mV with a Tafel slop of 64.3 mV dec^−1^ at a current density of 10 mA cm^−2^, which compares favorably against the NiCoMoS (132 mV with Tafel slop of 73.7 mV dec^−1^), NiCoS@HsGDY (197 mV with Tafel slop of 64.7 mV dec^−1^) and NiCoS (224 mV with Tafel slop of 72.5 mV dec^−1^). The enhanced HER performance of NiCoS@HsGDY over NiCoS points to the beneficial role of the HsGDY layers serving as selective electron and ion channel allowing electrolyte access to the inner layer. The further enhanced activity of NiCoS@HsGDY@Ni,Co-MoS_2_ compared to NiCoS@HsGDY indicates the importance of having three layers with different compositions and structures. In addition, the electrochemical impedance spectroscopy (EIS) spectra show that NiCoS@HsGDY@Ni,Co-MoS_2_ and NiCoS@HsGDY have smaller charge transfer resistance than NiCoMoS and NiCoS, respectively, at an overpotential of 200 mV (Fig. 7c). As known, the solution resistance *R*_s_ is independent of overpotential of HER, while the charge transfer resistance *R*_ct_ determines the electrocatalytic kinetics of HER. As displayed in the equivalent circuit diagrams (Supplementary Fig. 17), the values of *R*_ct_ decrease significantly from 10.2 Ω to 2.09 Ω when NiCoS is incorporated with HsGDY (NiCoS@HsGDY), suggesting much facilitated electron transfer by introducing HsGDY. A further decreased *R*_ct_ value of NiCoS@HsGDY@Ni,Co-MoS_2_ (0.638 Ω) clearly indicates the benefit of the triple-layer structure. When compared with NiCoMoS (0.748 Ω), the lower *R*_ct_ value of NiCoS@HsGDY@Ni,Co-MoS_2_ (0.638 Ω) also reveals the role of HsGDY as a built-in electron conductive channel during the HER process.

We further measure the specific surface area and the electrochemical active surface area (ECSA) of these nanoarray electrodes synthesized in this work. First, using cyclic voltammetry (CV) method, we determine the ECSA of NiCoS@HsGDY@Ni,Co-MoS_2_ and NiCoMoS by measuring the electrochemical double-layer capacitance (*C*_dl_), which is believed to be positively proportional to the ECSA^73^. As shown in the Supplementary Fig. 18, the *C*_dl_ of NiCoS@HsGDY@Ni,Co-MoS_2_ (75 mF cm^−2^) is larger than that of NiCoMoS (65.4 mF cm^−2^), indicating that the tri-layered nanostructure allows better electrolyte/products transfer in and out through the entire electrode than that of mixed NiCoMoS. The BET-specific surface areas of NiCoS@HsGDY@Ni,Co-MoS_2_, NiCoS@HsGDY, NiCoMoS, NiCoS, and carbon paper are 18.46, 16.75, 5.49, 3.17, and 0.75 m^2^ g^−1^, respectively (Supplementary Fig. 18), with the tri-layered nanostructure shows the highest surface area. Notably, an inner-connective porous structure is obvious in the interconnected nanosheets of Ni,Co-MoS_2_, especially at the interface between Ni,Co-MoS_2_ and HsGDY (red circle in Supplementary Fig. 19). Besides, both NiCoS@HsGDY@Ni,Co-MoS_2_ and HsGDY nanoarrays exhibit superhydrophilicity as shown in the Supplementary Fig. 20. Therefore, the electrolyte would permeate through the middle layer of microporous HsGDY, which acts as an ion channel that allows mass transfer through the micropores. Interestingly, the inner layer of NiCoS with HsGDY coating is also porous as shown in the blue circle in the Supplementary Fig. 19, which further permits the electrolyte into the hollowed-out cavity of the inner NiCoS layers. Therefore, the tri-layered nanoarrays in this work enable mass transfer through the entire electrode, which is desirable in HER and many other energy-related applications.

It is confirmed that HsGDY has no HER activity when the overpotential is smaller than 300 mV, which, when combined with the enhanced HER performance of NiCoS@HsGDY@Ni,Co-MoS_2_ against NiCoMoS, verifies its HER inactivity and the function of a built-in electron and ion channel in the triple-layered structure. Thus, we conclude that the triple-layered tubular texture with a unique built-in electron and ion channel allows for efficient chemical reaction kinetics.

Notably, a good HER performance with an overpotential of 100 mV dec^−1^ (10 mA cm^−2^) and Tafel slop of 89.5 mV dec^−1^ is achieved with tri-layered NiCoS@HsGDY@Ni,Co-MoS_2_, when performed in the alkaline solution (1 M KOH) (Fig. 7e and Supplementary Fig. 21). An overpotential of 160 mV at 10 mA cm^−1^ is also acquired in 1 M phosphate buffer solution (PBS) (pH = 7.1) (Fig. 7e and Supplementary Fig. 21). It has been reported that metal chalcogenides (e.g., MoS_2_) doped with secondary metal ions (e.g., Co, Ni) could be good candidates to perform HER in alkaline and neutral media with enhanced activity and stability^57,74^. The incorporation of heteroatoms is considered to promote the initial discharge of water to form the hydrogen intermediates (H_ad_) for HER in alkaline and neutral media, which consequently facilitates the generation of H_2_. Therefore, the incorporation of Ni and Co into MoS_2_ leads to the enhanced HER performance within a wide pH range. Besides, the stability of the core structure of Ni_3_S_2_/Co_9_S_8_ is also considerably promoted as a result of the seamlessly coated HsGDY layers.

As shown in Fig. 7d, f, the tri-layered NiCoS@HsGDY@Ni,Co-MoS_2_ nanoarrays exhibit stability with 96% retention of the initial current in 0.5 M H_2_SO_4_ (pH = 0.5) and 80% performance retention in 1 M PBS (pH = 7.1) after 10 h of HER. With 1 M KOH (pH = 14.1), there is an initial current-density decrease within the first 30 min, but a stable current persists afterward for 10 h of the HER. The SEM images show that the morphology of the tri-layered NiCoS@HsGDY@Ni,Co-MoS_2_ nanoarrays is well preserved under three pH conditions for 10 h of HER (Supplementary Fig. 22). It should be noted that although the middle HsGDY layers could effectively protect the core of Ni_3_S_2_/Co_9_S_8_ in alkaline electrolyte, a degree of reduction in mass transfer capability still occurred in the initial stages caused by the partial dissolution/aggregation of Ni_3_S_2_/Co_9_S_8_ (Supplementary Fig. 23). However, the built-in HsGDY layers lead to the long-term stability of the tri-layered NiCoS@HsGDY@Ni,Co-MoS_2_ nanoarrays beyond the initial stage. These results unequivocally reveal the stability of the tri-layered NiCoS@HsGDY@Ni,Co-MoS_2_ nanoarrays in this work. We further compare the HER stability of NiCoS@HsGDY@Ni,Co-MoS_2_, NiCoMoS, NiCoS@HsGDY and NiCoS for a duration of 10 h in 0.5 M H_2_SO_4_ (pH = 0.5). As shown in Fig. 7d, the well-aligned single-layered NiCoS nanoarrays are completely destroyed after 10 h HER process. However, the activity and stability of the core structure of Ni_3_S_2_/Co_9_S_8_ is considerably promoted in the presence of the HsGDY coating layer during HER (Supplementary Fig. 22). Besides, the HER performance of NiCoS@HsGDY@Ni,Co-MoS_2_ against NiCoMoS indicates the contribution of the core structure of NiCoS@HsGDY, which is otherwise destroyed without HsGDY protection after 2 h of HER (Fig. 7d). These results reveal the benefits of the built-in HsGDY layers to the HER performance of the electrode in both activity and stability.

The HER performance of NiCoS@HsGDY@Ni,Co-MoS_2_ is better than many of reported materials of 2H-MoS_2_, Co_9_S_8_, Ni_3_S_2_ (Supplementary Table 1). The effective HER performance of NiCoS@HsGDY@Ni,Co-MoS_2_ in a wide range of pH values is attributed to the following factors. First, the incorporation of Ni and Co in the Mo-S phase in the inner layer leads to multi-metallic sulfides with rich defects; secondly, the seamless sandwiched conductive HsGDY middle layer as a built-in electron and ion channel improves electrode kinetics owing to the large extended π-conjugated system; thirdly, the triple-layered tubular and edge-terminated texture and unique hierarchical porous nanostructure allows for exposure of more active sites and easy electrolyte permeation. Fourthly, the seamless and intimate contact between the HsGDY and both the inner and outer layers effectively prevent them from aggregation during the HER process. All of the above factors contribute to the excellent performance and durability of NiCoS@HsGDY@Ni,Co-MoS_2_, which shows well-preserved structure and performance after 10 h of HER evaluation. Therefore, we believe that these triple-layered nanostructures of HsGDY-sandwiched TMCs would be promising in other electrochemical applications, such as supercapacitors^75^, Na/Li ion storage^50^, oxygen evolution reaction^76^, CO_2_ reduction^77^, and water desalination^78^ etc.

## Discussion

In conclusion, this work reports a dual-template strategy that involves using HsGDY, one type of MONs, to coat a self-template of transition metals. Under suitable chemical reaction conditions,the dual template can lead to fabrication of triple-layered nanostructures in which each layer has a different chemical composition and nanostructure. Inert HsGDY is judiciously selected for this synthesis, as it physically confines the sacrificial template in place and simultaneously allows mass transfer through its micropores. Based on a dual-template of Ni-Co hydroxyl-carbonate nanoarays confined with a conformally coated HsGDY layer, triple-layered nanotube arrays composed of a HsGDY middle layer sandwiched between an external layer of Ni-,Co-co-doped MoS_2_ nanosheets and an internal layer of NiCoS (Co_9_S_8_, Ni_3_S_2_) nanotubular layer has been elaborately fabricated. The synthesis is versatile and grows the triple-layered nanostructures directly on conductive carbon papers, which makes readily it a suitable electrode material for many electrochemical applications. The triple-layered material delivers a HER performance better than most of the corresponding 2H-MoS_2_, Ni_3_S_2_ and Co_9_S_8_ over a wide range of pH values (0–14). This work contributes to the ongoing search for multi-layered nanostructures towards advanced energy conversion/storage applications.

## Methods

### Synthesis of (Ni,Co)(OH)_2_CO_3_ (NiCoHC) nanowire arrays

Ni-Co hydroxyl-carbonate nanowire arrays were directly grown on the carbon fibre paper according to a literature method^32^. Briefly, 0.95 g of CoCl_2_·6H_2_O, 0.475 g of NiCl_2_·6H_2_O, 0.43 g of urea, and 0.58 g of cetyltrimethylammonium bromide (CTAB) were dissolved into 40 mL of deionized water under sonication to form a clear pink solution. Then, the solution was transferred to a 50 mL Teflon-lined stainless steel autoclave containing the precleaned carbon paper (FuelCellsEtc, AvCarb MGL370, 2.5 × 6 cm^2^, see Supplementary Information for details). After hydrothermal treatment at 100 °C for 10 h in an oven, the carbon paper with NiCoHC nanowire arrays was taken out of the autoclave, and then cleaned by ultrasonication with DI water and ethanol repeatedly to remove the loosely attached products.

### Synthesis of core-shell nanowire arrays

A conformal layer of HsGDY was directly coated on the surface of the NiCoHC nanowire arrays. In details, the carbon paper with NiCoHC nanowire arrays on its surface (1 × 6 cm^2^) was fixed in a 100 mL pear-shaped flask containing tetrahydrofuran (20 mL) and trimethylamine (40 mL) with catalysts of Pd(PPh_3_)_2_Cl_2_ (16.8 mg) and CuI (4.4 mg). After stirring at room temperature for 30 mins under argon atmosphere, 1,3,5-triethynylbenzene (10 mg) was added. Then the reactor was sealed and heated at 60 °C for 24 h under argon atmosphere. Microporous HsGDY networks gradually generated on the surface of the nanowire arrays through Glaser coupling of 1,3,5-triethynylbenzene. The conformal NiCoHC@HsGDY nanowire arrays with a color of brown was obtained by ultrasonication with ethanol.

### Synthesis of tri-layered nanotube arrays

Then, the as-prepared NiCoHC@HsGDY nanoarrays were utilized as a dual template to construct triple-layered nanostructures by the reaction with (NH_4_)_2_MoS_4_ in the presence of N_2_H_4_. In details, the carbon paper with the surface NiCoHC@HsGDY nanowire arrays (1 × 2 cm^2^) was placed in an autoclave containing 10 mL DMF, 10 mg (NH_4_)_2_MoS_4_, and 30 μL N_2_H_4_ under the protection of Argon gas. The autoclave was subjected to 15 h 210 °C hydrothermal process, by the end of which the triple-layered NiCoS@HsGDY@Ni,Co-MoS_2_ nanotube arrays with a color of black was acquired. The carbon paper was then sonicated with water and ethanol to remove the loosely attached products. For the purpose of confirmation, triple-layered nanoarrays of Co_9_S_8_@HsGDY@Co-MoS_2_, and nanosheet arrays of Ni_3_S_2_@HsGDY@Ni-MoS_2_ were also synthesized when Co(OH)F@HsGDY nanowire arrays and Ni(OH)F@HsGDY nanosheet arrays were used as dual-template with other conditions being all the same (see Supplementary Information). NiCoS and NiCoS@HsGDY nanoarrays were further prepared purposefully to verify the HER performance of the core structures and reveal the function of built-in HsGDY layers (see Supplementary Information).

### Characterization

The SEM images were taken with a Zeiss Merlin scanning electron microscope. TEM, high-resolution TEM (HRTEM), HAADF-STEM images, EELS, and STEM-EELS elemental mapping were obtained with a ThermoFisher Scientific’s Titan ST equipped with a Gatan Image Filter (GIF) Tridiem. The ultrathin sections (70 nm) cured by epoxy resin were prepared on an Ultramicrotome with a glass blade and then placed on a carbon-coated copper grid followed by TEM imaging. The XRD patterns of the products were recorded with Bruker D8 ADVANCE Diffraction System using a Cu Kα source (*λ* = 1.5406 Å). Raman spectra was collected on a Horiba Aramis with a 473 nm laser wavelength excitation and spectra were up to 3000 cm^−1^. XPS was carried out on an Axis Ultra instrument (Kratos Analytical) under ultrahigh vacuum (<10^−9^ mbar) by using a monochromatic Al Kα X-ray source (hυ = 1486.6 eV) operated at 150 W. Solid NMR spectra were acquired using WB Bruker 600 AVANAC III spectrometer equipped with 2.5 mm double resonance MAS Bruker Probe (BrukerBioSpin, Rheinstetten, Germany). The surface area and pore size distributions of the synthesized materials were determined by a Micromeeitics-TriStar II system. The surface area was calculated using the BET method and the pore size distributions were calculated using the Density Functional Theory (DFT) from the desorption branch. The Fourier transform infrared spectroscopy (FTIR) was recorded on a FTIR-is10 spectrometer using a diamond. See Supporting Information for the details of XPS and solid NMR. Static contact angles were conducted with a commercial contact angle system (OCA 35, DataPhysics, Filderstadt, Germany) to quantify the hydrophilicity of the synthesized nanostructures.

### Electrocatalytic HER

Electrochemical measurements were carried out in a three-electrode system consisting of a working electrode, a graphit rod counter electrode, and an Ag/AgCl (3 M KCl) reference electrode conducted with Autolab PGSTAT 302 N potentiostat (Metrohm Autolab, Netherlands). All the measured potentials were referred to reverse hydrogen electrode (RHE) with the equation of *E*(RHE) = *E*(Ag^+^/AgCl) + 0.210 V + 0.0591 pH. The aqueous solution of 0.5 M H_2_SO_4_ (pH = 0.5), 1 M phosphate buffer solution (PBS, pH = 7.1) and 1 M KOH (pH = 14.1) were utilized to evaluate their HER performance over a wide pH values. The linear sweep voltammetry (LSV) for water reduction was carried out at a scan rate of 5 mV s^−1^. The polarization curves were plotted as overpotential (*η*) verse log current (log *j*) which was used to evaluate the Tafel slopes by fitting the linear portion to the Tafel equation (*η* *=* b log*j* *+* a). All polarization curves were *iR* corrected. The EIS was performed at the overpotential of 200 mV from 100 kHz to 10 mHz. The time-dependent current density (I-t) curves were measured at overpotential of 200 mV.

### Data availability

The data that support the findings of this study are available from the corresponding authors on request.

## Electronic supplementary material

Supplementary Information
