# Supplementary material for: Dual-template engineering of triple-layered nanoarray electrode of metal chalcogenides sandwiched with hydrogen-substituted graphdiyne
Source: Nat Commun. 2018 Aug 7;9:3132. doi: 10.1038/s41467-018-05474-0 (PMC6081434; doi:10.1038/s41467-018-05474-0)
Supplement: Supplementary file 1 — Supplementary Information [file 41467_2018_5474_MOESM1_ESM.docx]

**Dual-template engineering of triple-layered nanoarray electrode of metal chalcogenides sandwiched with hydrogen-substituted graphdiyne**

Sifei et al.

**
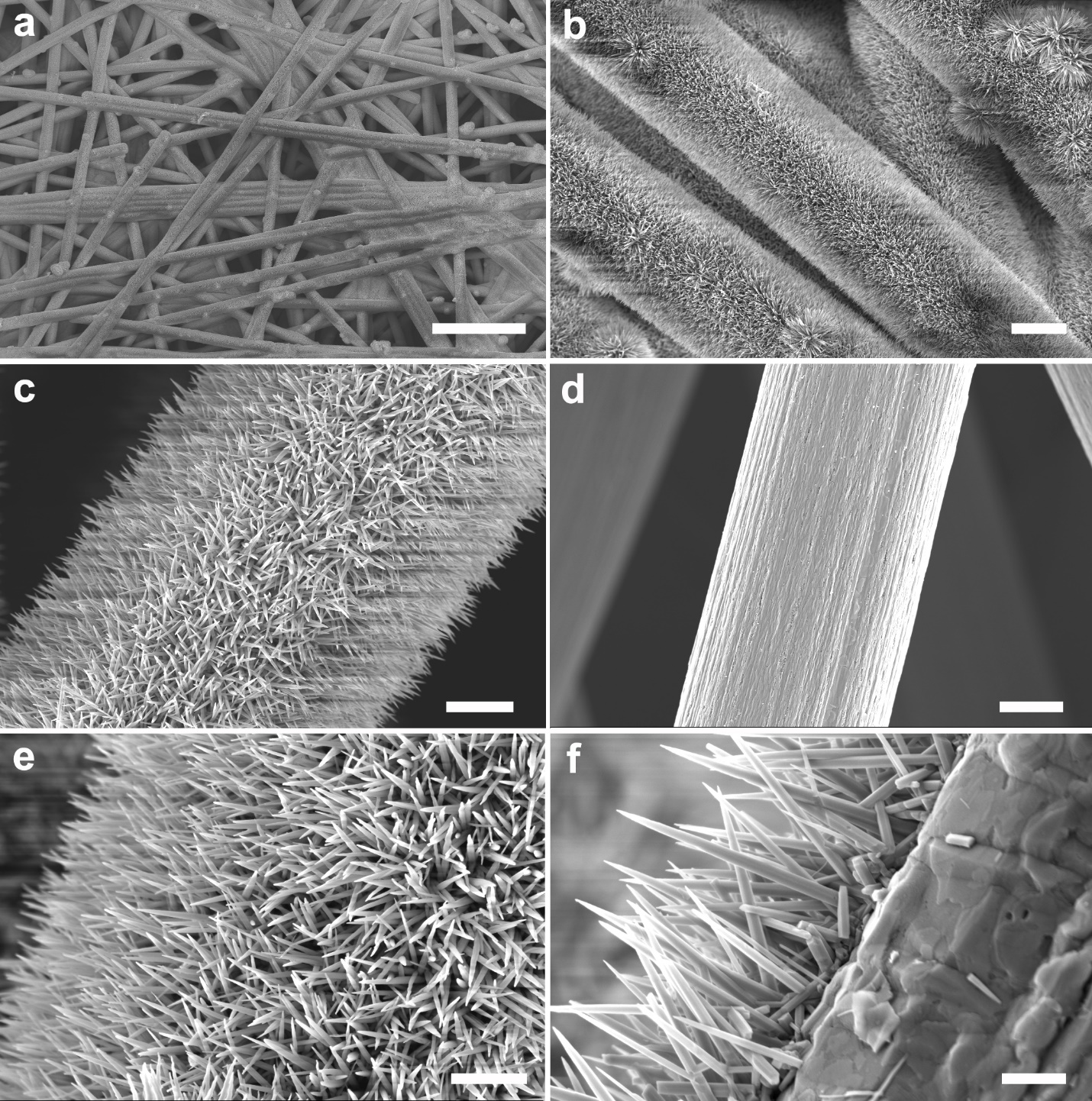
**

**Supplementary Figure 1** Scanning electron microscopy micrographs. **a-c**, **e**, **f** Scanning electron microscopy (SEM) images of well-aligned and tapering Ni-Co hydroxyl-carbonate nanowire arrays with diameter of around 100-200 nm and length up to 2 μm, which are uniformly grown around the cylindrical carbon fibres of a commercial carbon paper, and **d** the corresponding cleaned and unmodified carbon fibre. Scale bars: (**a**) 100 μm, (**b**) 5 μm, (**c, d**) 2 μm, (**e**) 1 μm, (**f**) 500 nm.

**
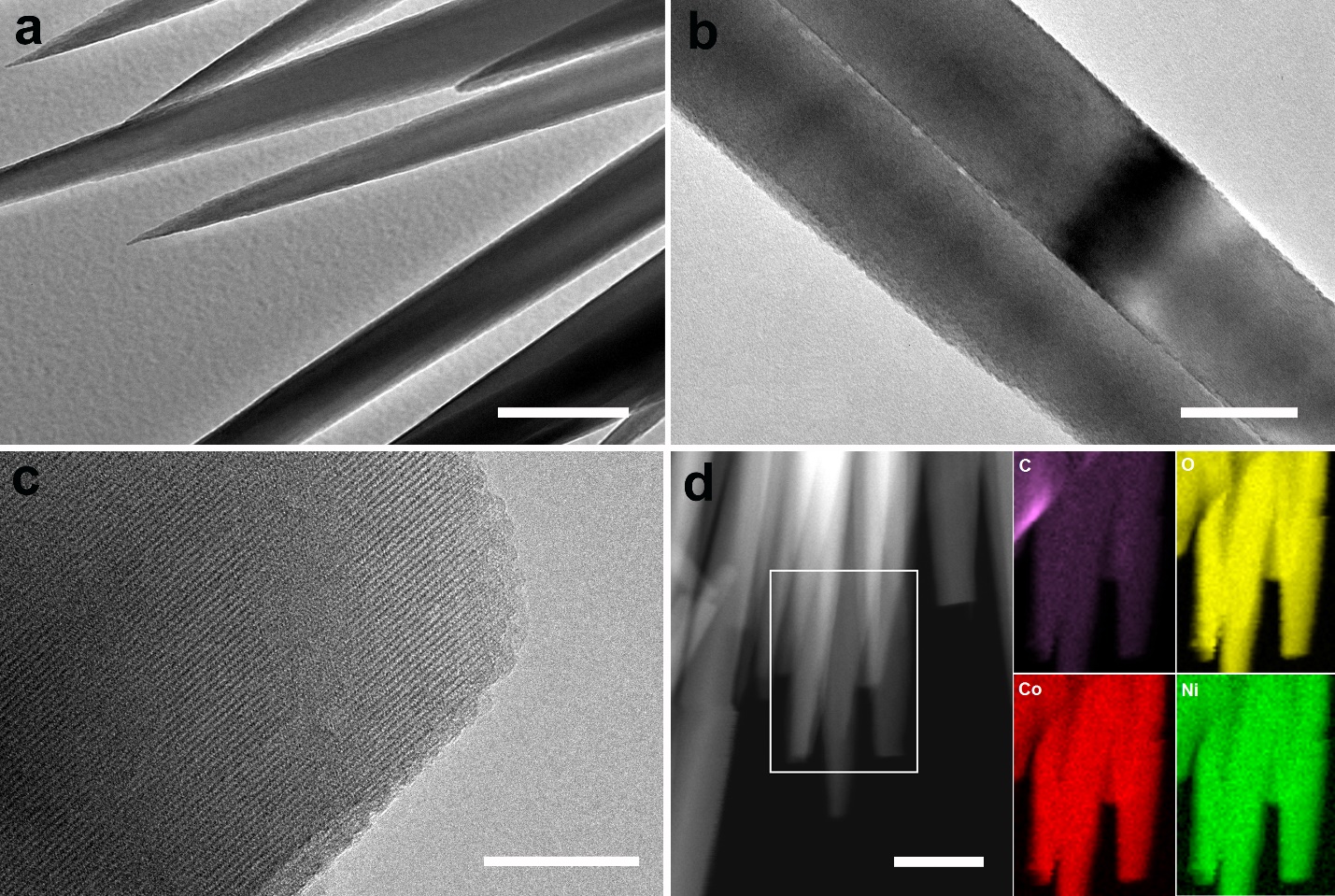
**

**Supplementary Figure 2** Transmission electron microscopy micrographs. **a, b** transmission electron microscopy (TEM) images, **c** high-resolution transmission electron microscopy (HRTEM) image and **d** scanning transmission electron microscopy-energy loss spectroscopy (STEM-EELS) elemental mappings of the as-prepared (Ni,Co)(OH)_2_CO_3_ (NiCoHC) nanowires. These results clearly indicate the solid nature of these nanowires with bimetallic character with single crystalline. Scale bars: (**a, d**) 200 nm, (**b**) 100 nm, (**c**) 20 nm.


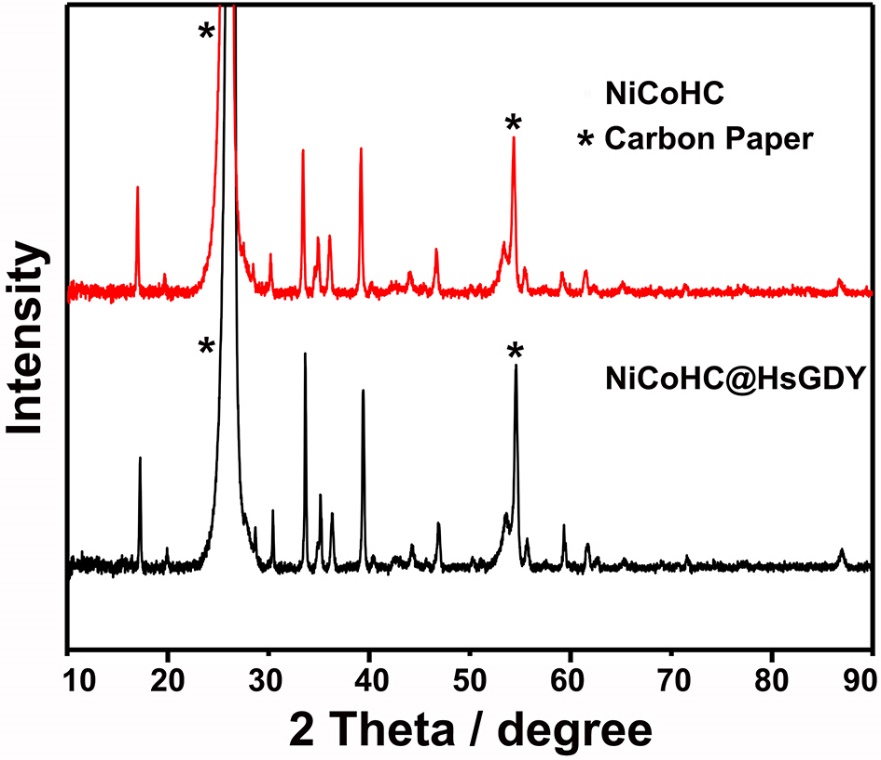


**Supplementary Figure 3** X-ray diffraction patterns. X-ray diffraction (XRD) patterns of (Ni,Co)(OH)_2_CO_3_ (NiCoHC) nanowires and NiCoHC@hydrogen-substituted graphdiyne (HsGDY) nanowire arrays directly grown on the carbon fibre paper. The consistent XRD patterns verify the encapsulated solid nanowires as Ni-Co hydroxyl-carbonate without any change before and after the HsGDY coating, while the HsGDY layers have amorphous feature.

**
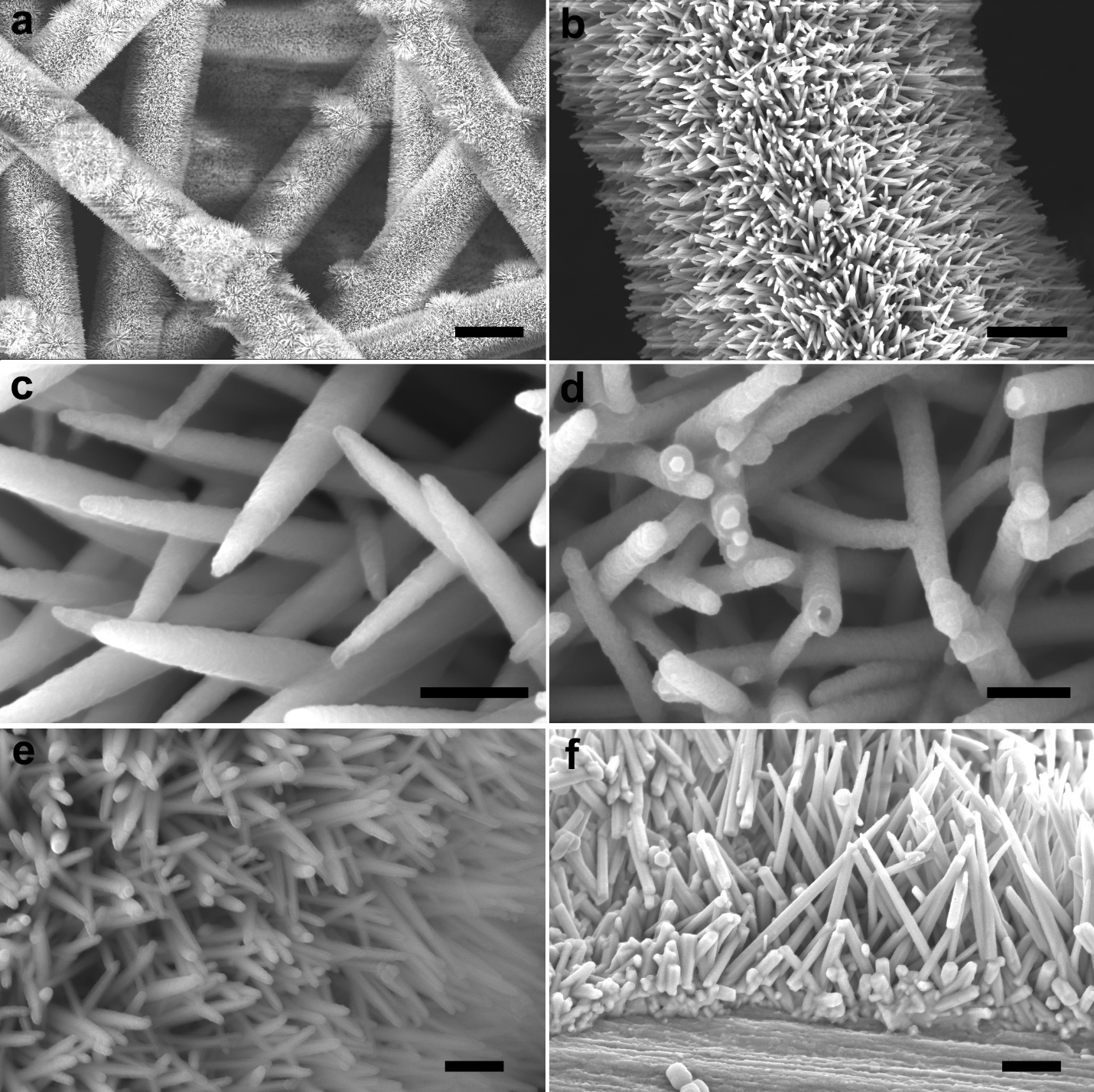
**

**Supplementary Figure 4** Scanning electron microscopy micrographs. **a-f** Scanning electron microscopy (SEM) images of the dual-template of (Ni,Co)(OH)_2_CO_3_ (NiCoHC) nanowire arrays conformally coated with hydrogen-substituted graphdiyne (HsGDY) layers. Scale bars: (**a**) 10 μm, (**b**) 2 μm, (**c, d**) 200 nm, (**e, f**) 500 nm.

**
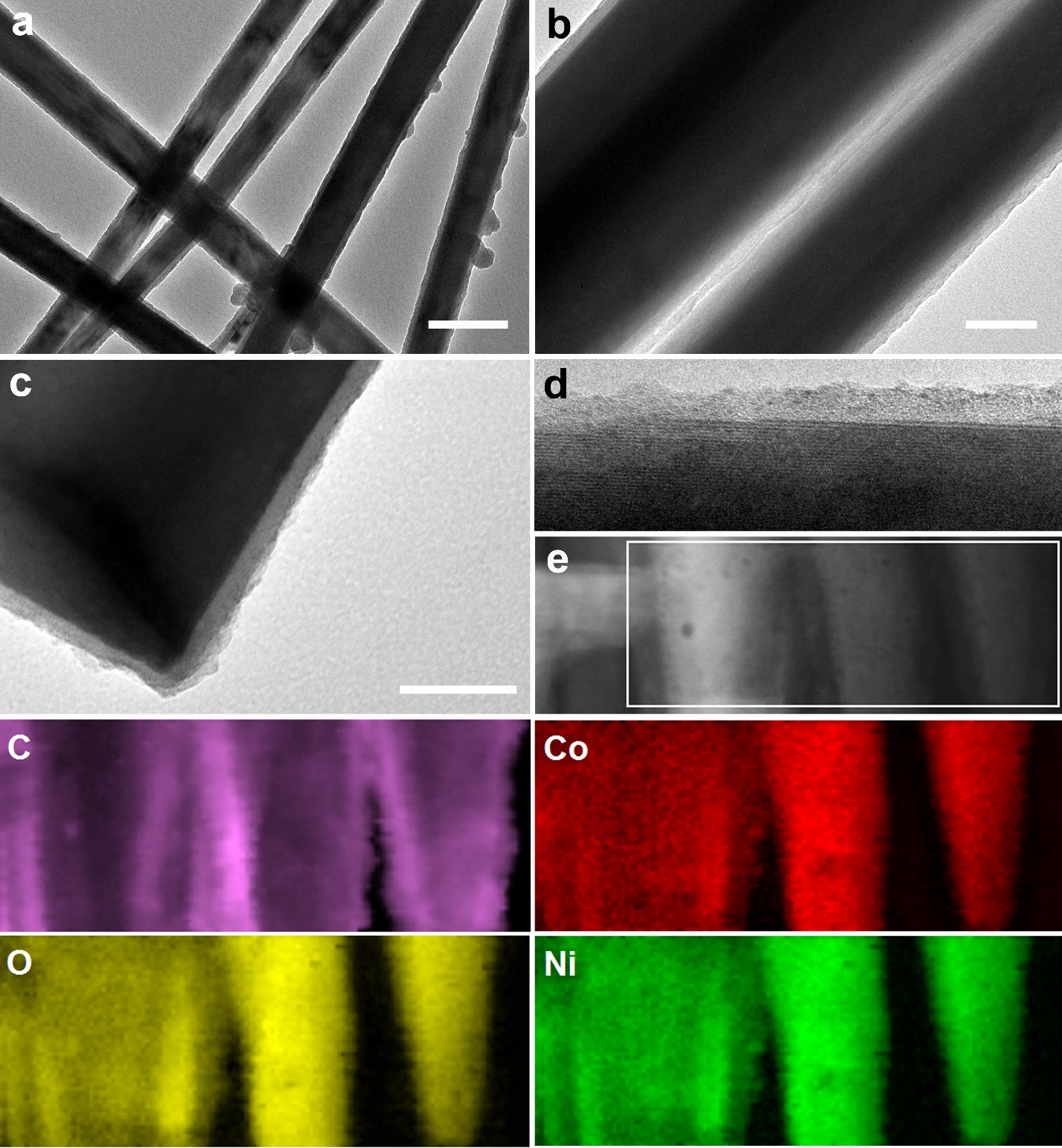
**

**Supplementary Figure 5** Transmission electron microscopy micrographs. **a-c** transmission electron microscopy (TEM) images, **d** high magnification TEM image and **e** scanning transmission electron microscopy-energy loss spectroscopy (STEM-EELS) elemental mappings of the dual-template of (Ni,Co)(OH)_2_CO_3_@hydrogen-substituted graphdiyne (NiCoHC@HsGDY) nanowires. Scale bars: (**a**) 300 nm, (**b**) 100 nm, (**c**) 50 nm.


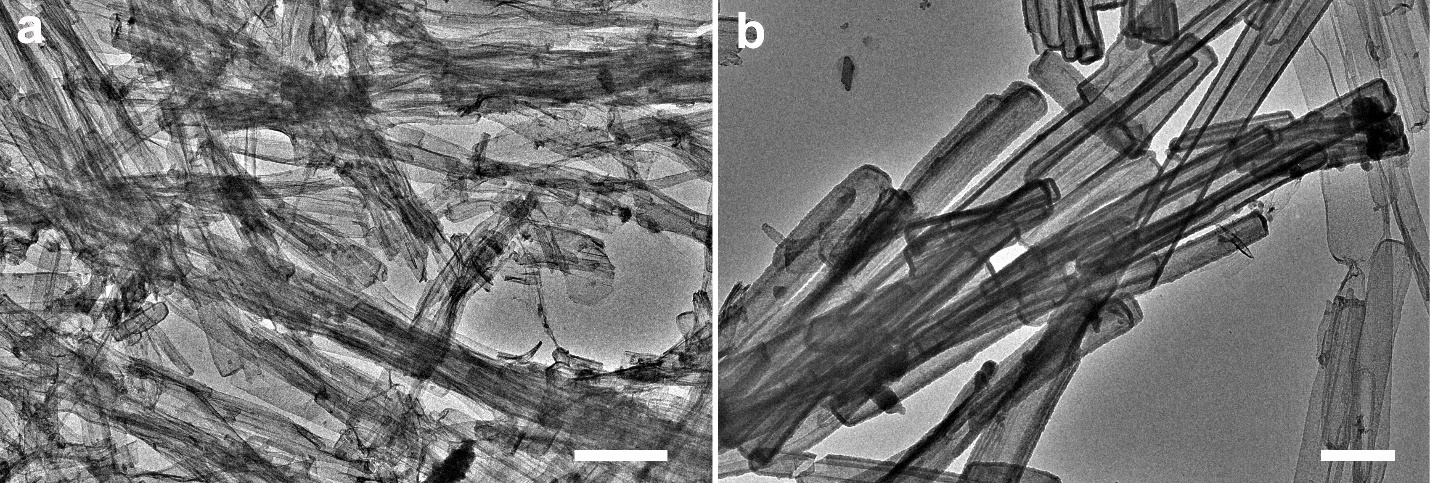


**Supplementary Figure 6** Transmission electron microscopy micrographs. **a, b** transmission electron microscopy (TEM) images of the discrete hydrogen-substituted graphdiyne (HsGDY) nanotubes by removing the interior self-template of (Ni,Co)(OH)_2_CO_3_ nanowires with 0.1 M HCl aqueous solution. Scale bars: (**a**) 1 μm, (**b**) 500 nm.


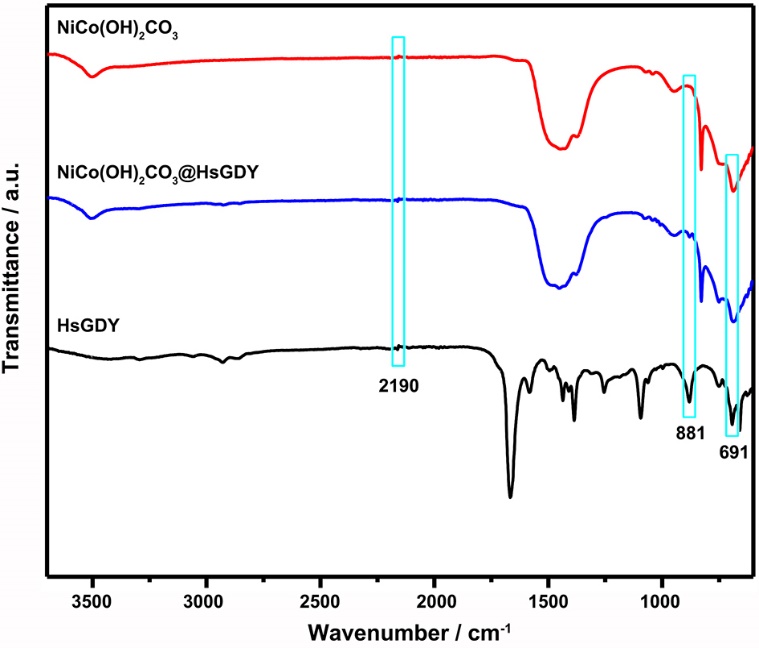


**Supplementary Figure 7** Fourier transform infrared spectra. Fourier transform infrared (FTIR) spectra of (Ni,Co)(OH)_2_CO_3_ nanowires, (Ni,Co)(OH)_2_CO_3_@hydrogen-substituted graphdiyne (HsGDY) nanowires and HsGDY nanotubes. The HsGDY nanotubes are obtained by removing the (Ni,Co)(OH)_2_CO_3_ of (Ni,Co)(OH)_2_CO_3_@HsGDY nanowires using acid. The characteristic peaks of the C≡C (2190 cm^-1^) and the 1,3,5-substituted benzene (881 and 691 cm^-1^) verify the structure of HsGDY as (poly(phenylenebutadiynylene)s and also confirm the successful coating of (Ni,Co)(OH)_2_CO_3_ nanowires with HsGDY.

**
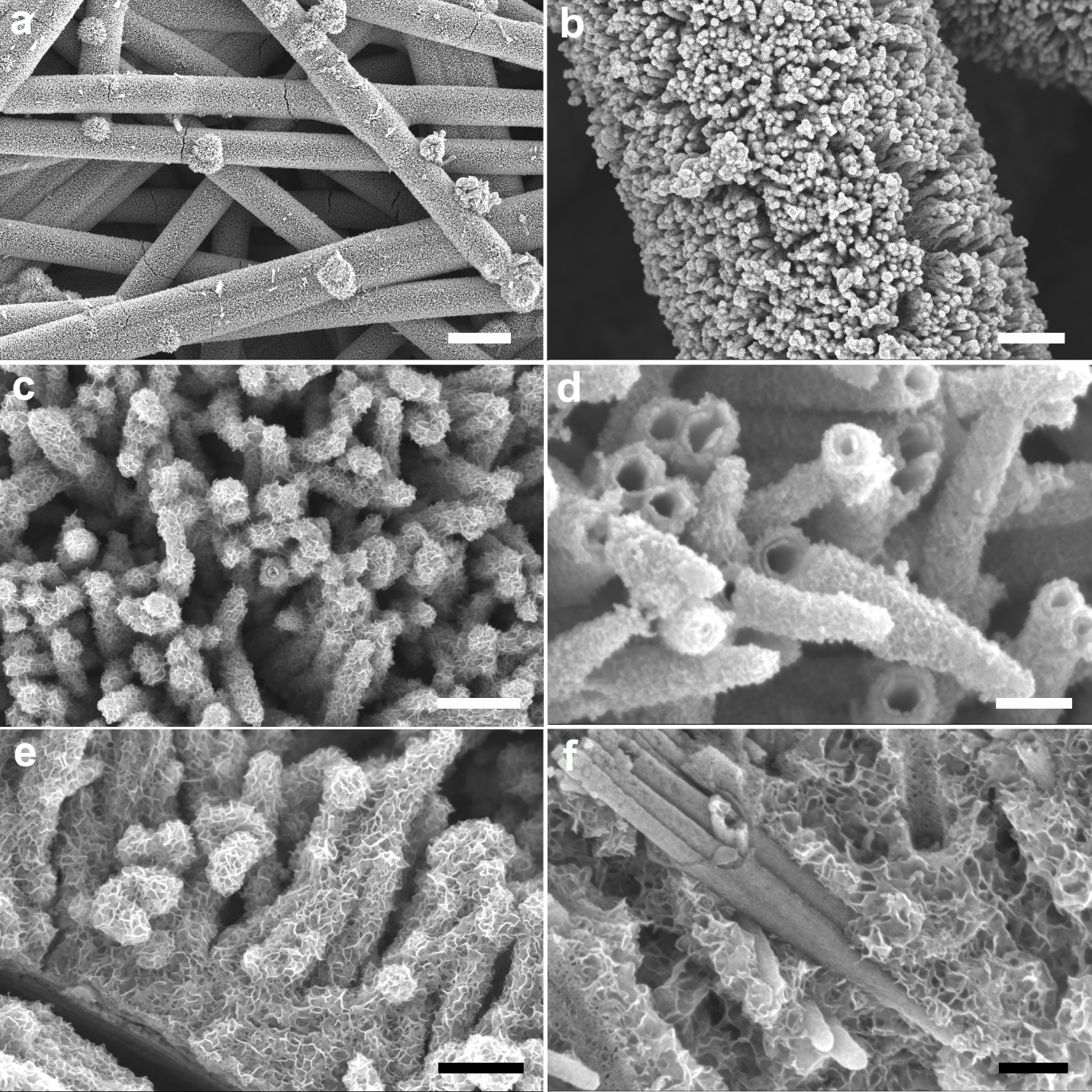
**

**Supplementary Figure 8** Scanning electron microscopy micrographs. **a-f** scanning electron microscopy (SEM) images of the as-prepared triple-layered tube-in-tube NiCoS@HsGDY@Ni,Co-MoS_2_ nanoarrays comprised of Ni_3_S_2_/Co_9_S_8_ inner layer (NiCoS), hydrogen-substituted graphdiyne (HsGDY) and Ni-,Co-co-doped MoS_2_ (Ni,Co-MoS_2_). High magnification SEM images (**d-f**) taken from broken areas reveal the formation of triple-layered tube-in-tube nanostructure. Scale bars: (**a**) 20 μm, (**b**) 2 μm, (**c, e**) 500 nm, (**d, f**) 200 nm.

**
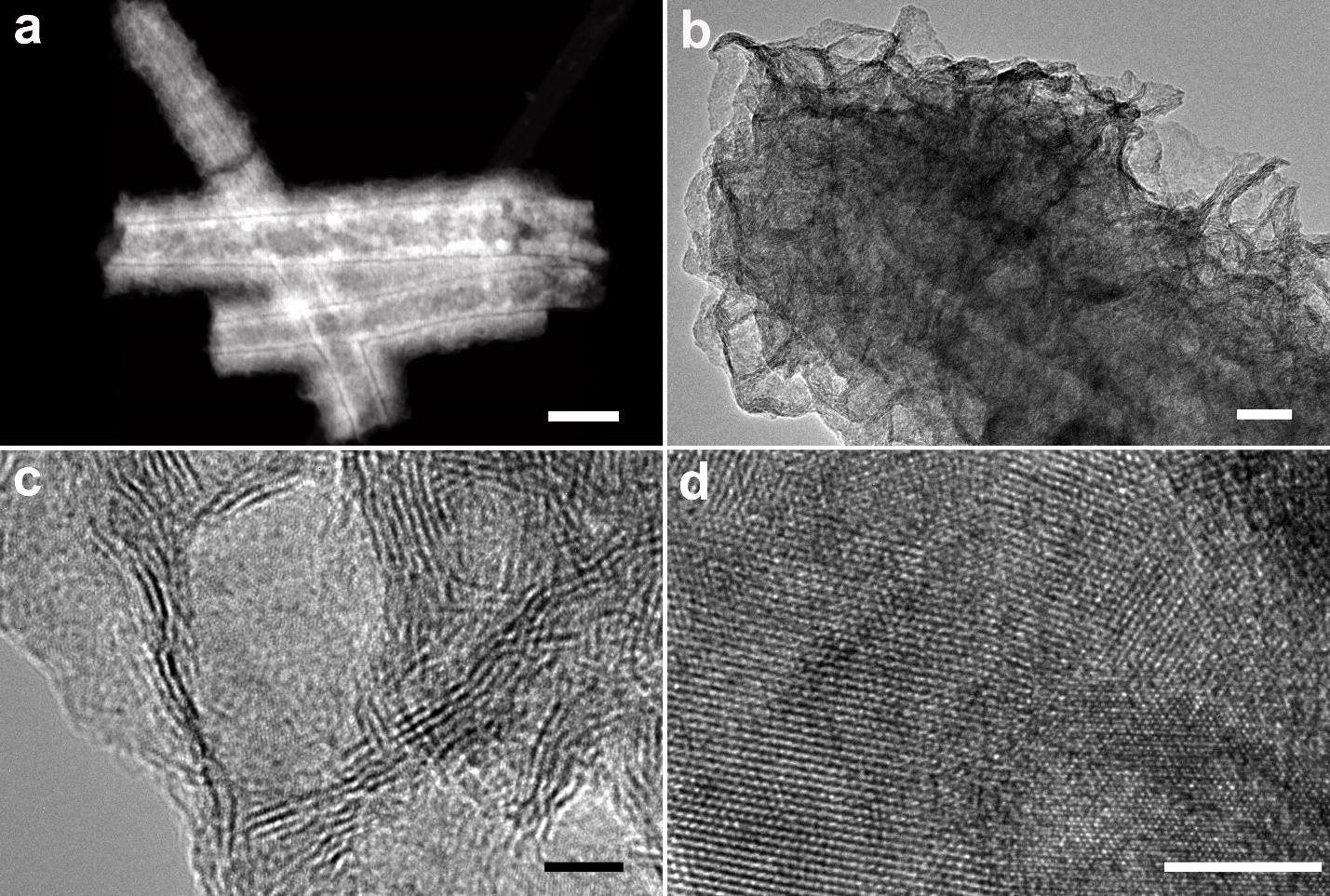
**

**Supplementary Figure 9** Transmission electron microscopy micrographs. **a** high angle annular dark field-scanning transmission electron microscopy (HAADF-STEM) image and **b** TEM image of the triple-layered tube-in-tube NiCoS@HsGDY@Ni,Co-MoS_2_ nanotubes comprised of Ni_3_S_2_/Co_9_S_8_ inner layer (NiCoS), hydrogen-substituted graphdiyne (HsGDY) and Ni-,Co-co-doped MoS_2_ (Ni,Co-MoS_2_). **c** HRTEM images of the outer layer of Ni,Co-MoS_2_ nanosheets and **d** the inner layer of NiCoS (Co_9_S_8_ and Ni_3_S_2_) nanotubes. Scale bars: (**a**) 100 nm, (**b**) 20 nm, (**c, d**) 5 nm.


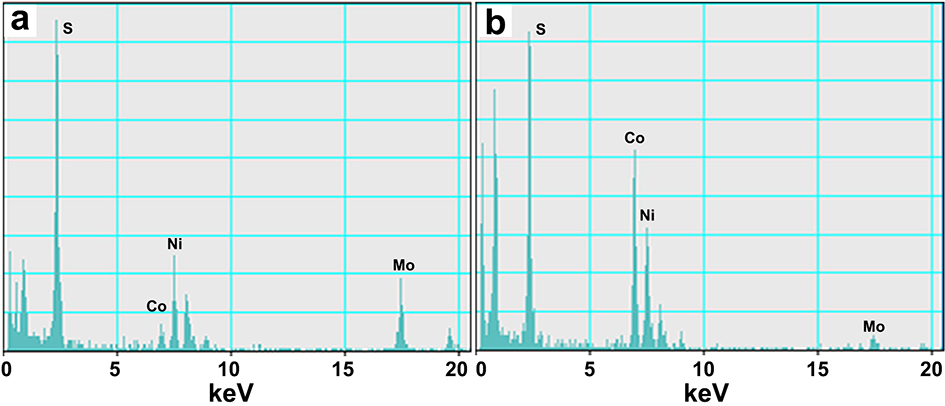


**Supplementary Figure 10** Energy dispersive spectroscopy spectra. **a, b** energy dispersive spectroscopy (EDS) spectra collected at the Ni-, Co-co-doped MoS_2_ (Ni,Co-MoS_2_) nanosheet outer layer (**a**) and the Ni_3_S_2_/Co_9_S_8_ (NiCoS) nanotube inter layer (**b**).


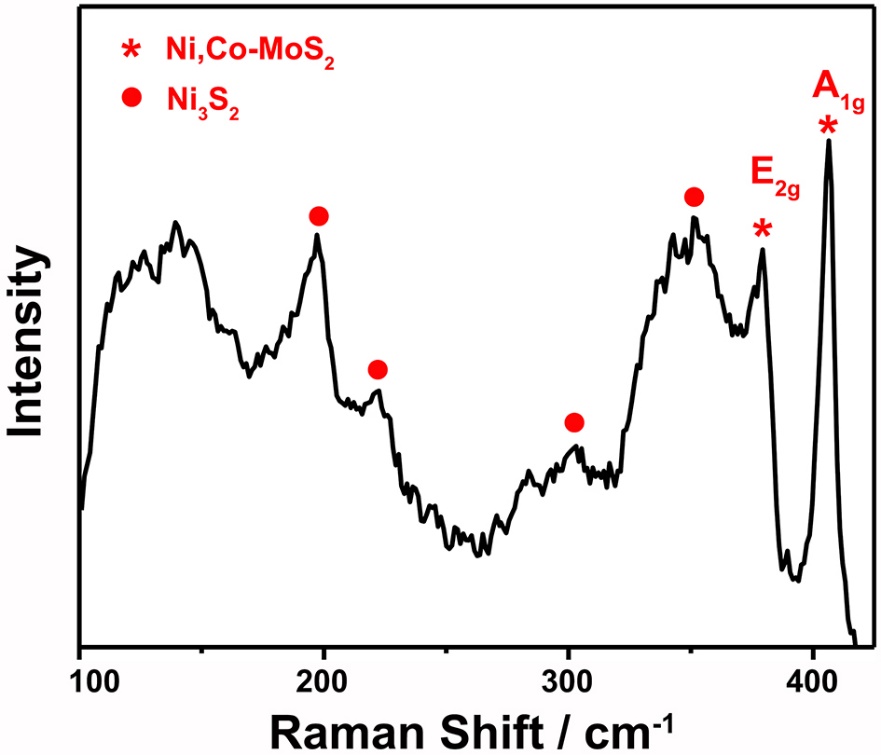


**Supplementary Figure 11** Raman spectrum of the triple-layered NiCoS@HsGDY@Ni,Co-MoS_2_ comprised of Ni_3_S_2_/Co_9_S_8_ inner layer (NiCoS), hydrogen-substituted graphdiyne (HsGDY) and Ni-,Co-co-doped MoS_2_ (Ni,Co-MoS_2_). The characteristic Raman bands at 196, 211, 302, 351 cm^-1^ are related to Ni-S bonds of Ni_3_S_2_. The Raman bands at 379 and 401 cm^-1^ correspond to the in-plane of E_2g_ and out-of-plane of A_1g_ modes of hexagonal MoS_2_, indicating the similar layered structure of Ni,Co-MoS_2_ to pure MoS_2._


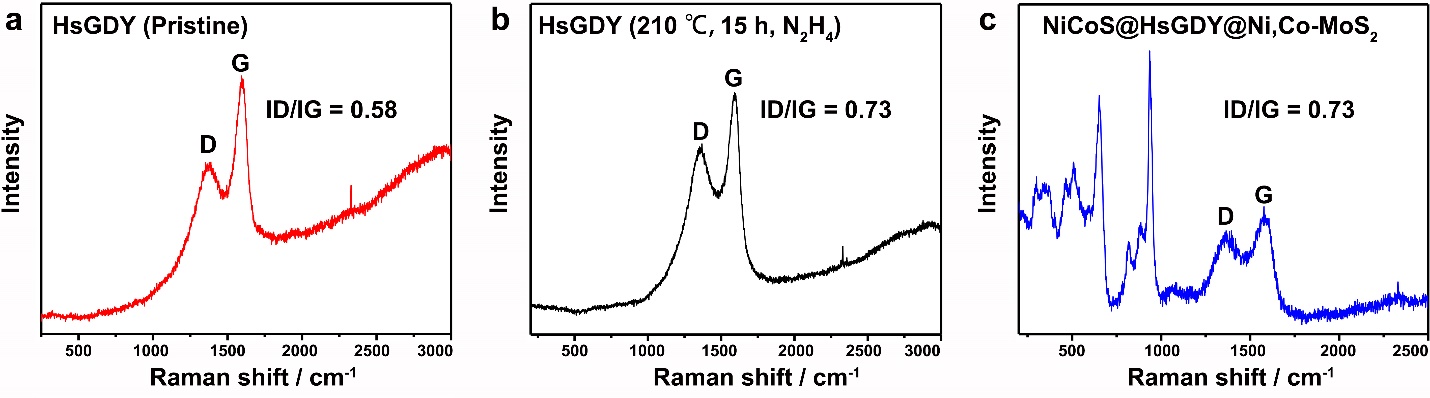


**Supplementary Figure 12** **a, b** Raman spectra of the discrete HsGDY nanotubes before (**a**) and after (**b**) treatment in DMF solvent in the presence of N_2_H_4_ at 210 ℃ for 15 hours; **c** Raman spectrum of tri-layered NiCoS@HsGDY@Ni,Co-MoS_2_ nanoarrays comprised of Ni_3_S_2_/Co_9_S_8_ inner layer (NiCoS), hydrogen-substituted graphdiyne (HsGDY) and Ni-,Co-co-doped MoS_2_ (Ni,Co-MoS_2_).

**Supplementary Note 1**

The increased ID/IG values of HsGDY after the chemical transformation is attributed to the defects formed in the HsGDY during the solvothermal treatment process. In order to verify this, Raman spectra of the HsGDY before and after solvothermal treatment are measured and compared to identify the influence of solvothermal environment on the variation in ID/IG values of HsGDY during the chemical transformation process. For the purpose of identifying the influence of solvothermal process on the variation in ID/IG values of HsGDY, pure and pristine HsGDY nanotubes are synthesized (Supplementary Fig. 6) whose ID/IG value is determined to be 0.58 (Supplementary Fig. 12a). These discrete HsGDY nanotubes are then subjected to the same treatment in DMF solvent in the presence of N_2_H_4_ at 210 ℃ for 15 hours. Consequently, the ID/IG value of the HsGDY sample after the treatment increases to 0.73 (Supplementary Fig. 12b), indicating the formation of some defects in HsGDY nanotubes during the solvothermal process. Furthermore, the same ID/IG value of 0.73 for the tri-layered NiCoS@HsGDY@Ni,Co-MoS_2_ (with the same solvothermal process) further verifies that the increased ID/IG value was a result of the defects generated in the HsGDY layers during the solvothermal process.


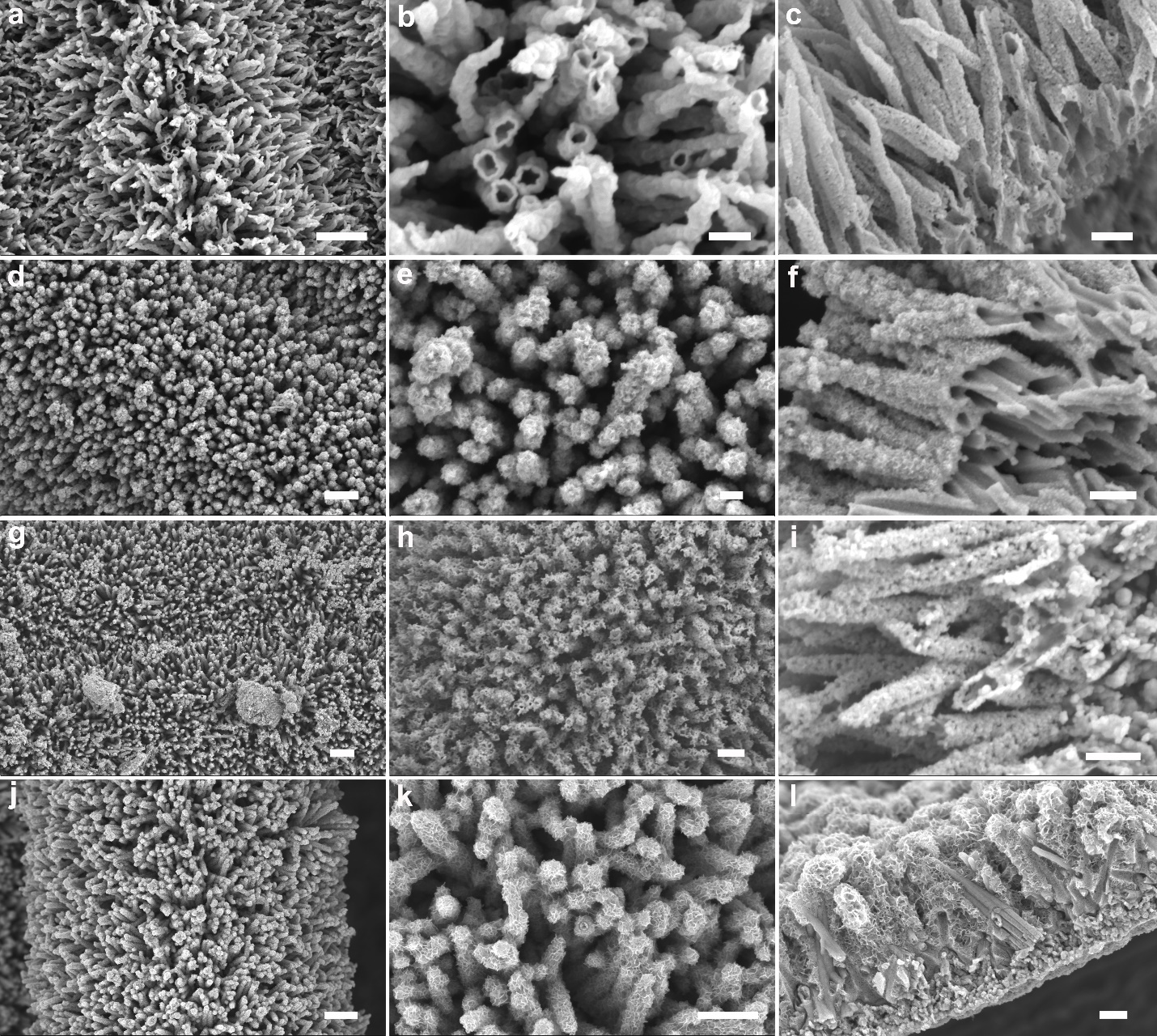


**Supplementary Figure 13 a-l** Scanning electron microscopy micrographs. SEM images of (Ni_x_Co_1-x_)MoS_4_ nanotube arrays (**a-c**) and NiCoMoS (Ni_3_S_2_, Co_9_S_8_, Ni,Co-MoS_2_) nanotube arrays (**d-f**) prepared by reaction the self-template of NiCoHC with (NH_4_)_2_MoS_4_ with the absence and presence of N_2_H_4_, respectively. SEM images of NiCoMoS ((Ni_x_Co_1-x_)MoS_4_/HsGDY) nanotube arrays (**g-i**) and NiCoS ((Ni_3_S_2_/Co_9_S_8_)@HsGDY@Ni,Co-MoS_2_) nanotube arrays (**j-l**) prepared by the dual-template of NiCoHC@HsGDY reacting with (NH_4_)_2_MoS_4_ in the absence and presence of N_2_H_4_, respectively. As shown in the figure **g-i**, (Ni_x_Co_1-x_)MoS_4_ nanoparticles are generated around the external surface of HsGDY while there is no any nanostructure in the interior of HsGDY, confirming the critical role of N_2_H_4_ in the growth of Ni,Co-MoS_2_ on the external surface of HsGDY. On the one hand, the produced H_2_S could diffuse into the interior of HsGDY to react with NiCoHC to form NiCoS (Co_9_S_8_, Ni_3_S_2_) in the presence of N_2_H_4_. On the other hand, some Ni^2+^ and Co^2+^ ions could diffuse outward to participate in the formation of MoS_2_ nanosheets to form Ni,Co-MoS_2_. Scale bars: (**a, d, g, j**) 1 μm, (**b, e**) 200 nm, (**h, k**) 500 nm, (**c, f, i, l**) 300 nm.

**
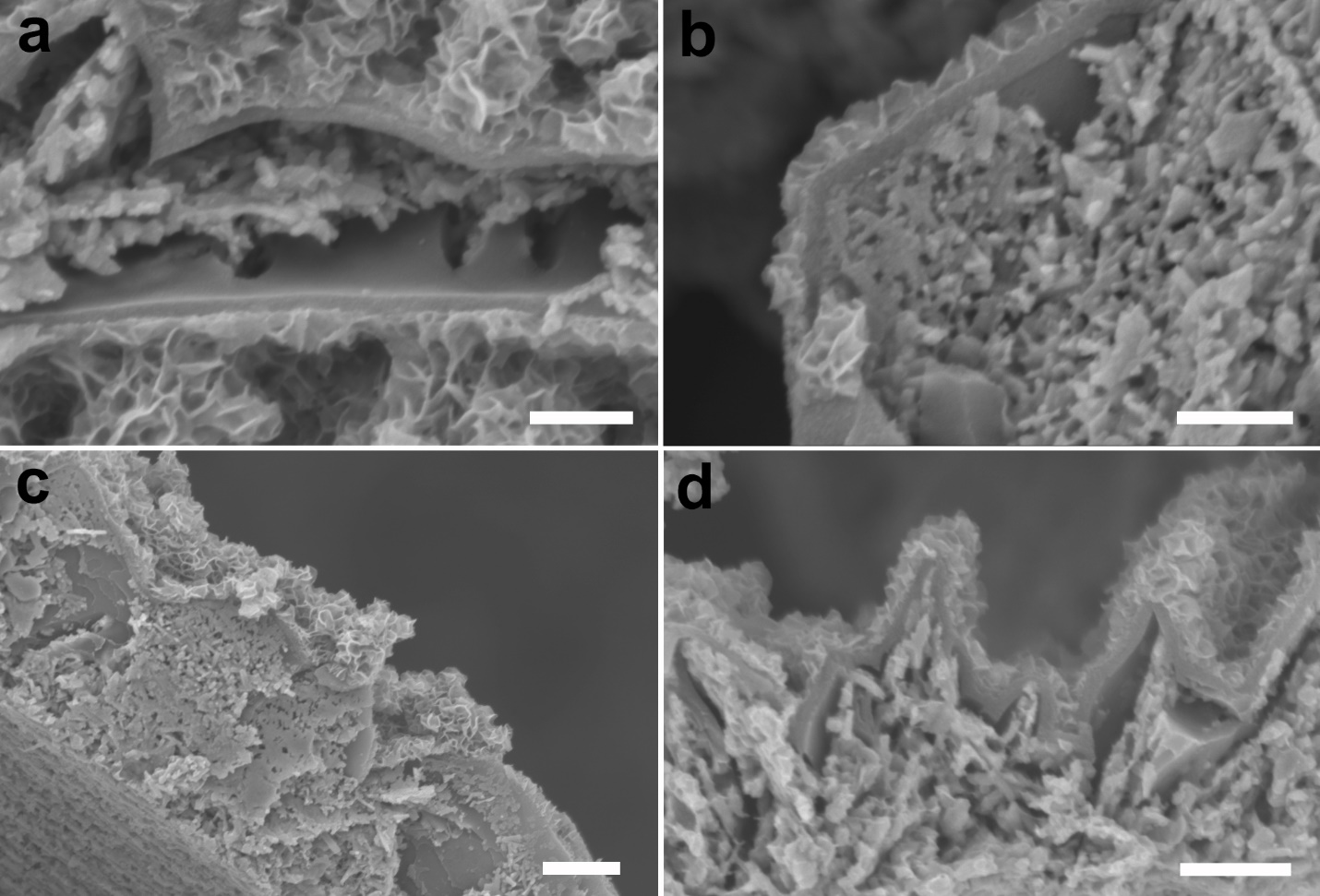
**

**Supplementary Figure 14** Scanning electron microscopy micrographs. **a-d** Scanning electron microscopy micrographs (SEM) images of Ni_3_S_2_@HsGDY@Ni-MoS_2_ taken from some broken areas. As can be seen, there are flower-like nanosheets seamlessly grown on the HsGDY layers with clear-cut and continuous layer boundaries. Meanwhile, some nanoparticle-assembled hollow nanosheets are also observed. Scale bars: (**a, b, d**) 200 nm, (**c**) 400 nm.


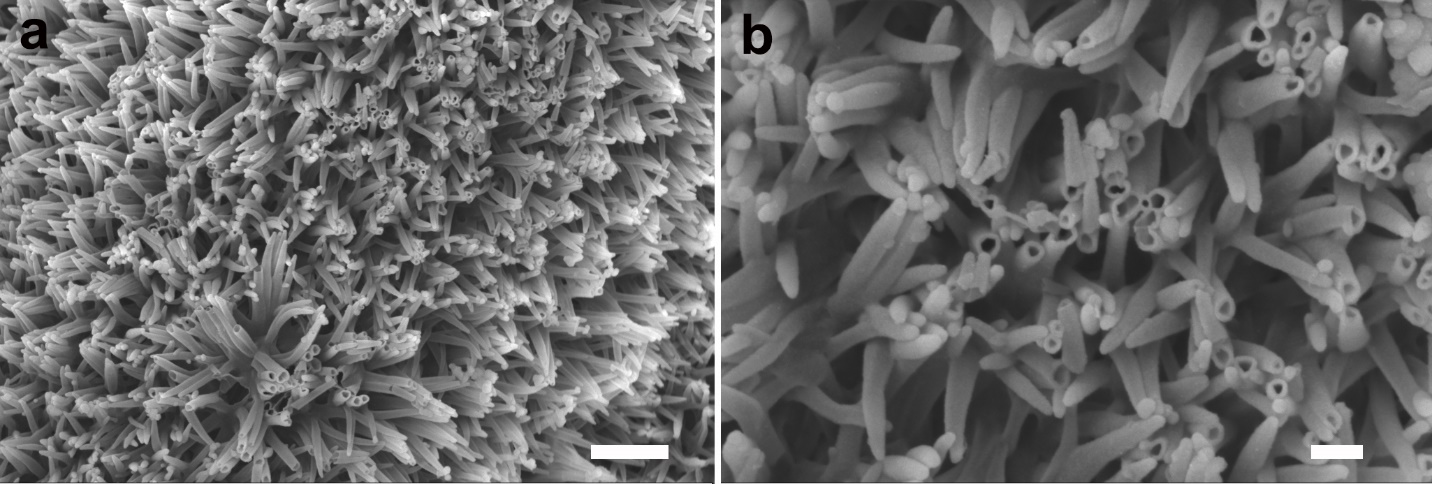


**Supplementary Figure 15** Scanning electron microscopy micrographs. **a, b** Scanning electron microscopy (SEM) images of the hydrogen-substituted graphdiyne (HsGDY) nanotube arrays after removing the interior self-template of NiCoHC with 0.1 M HCl aqueous solution. Scale bars: (**a**) 1 μm, (**b**) 200 nm.


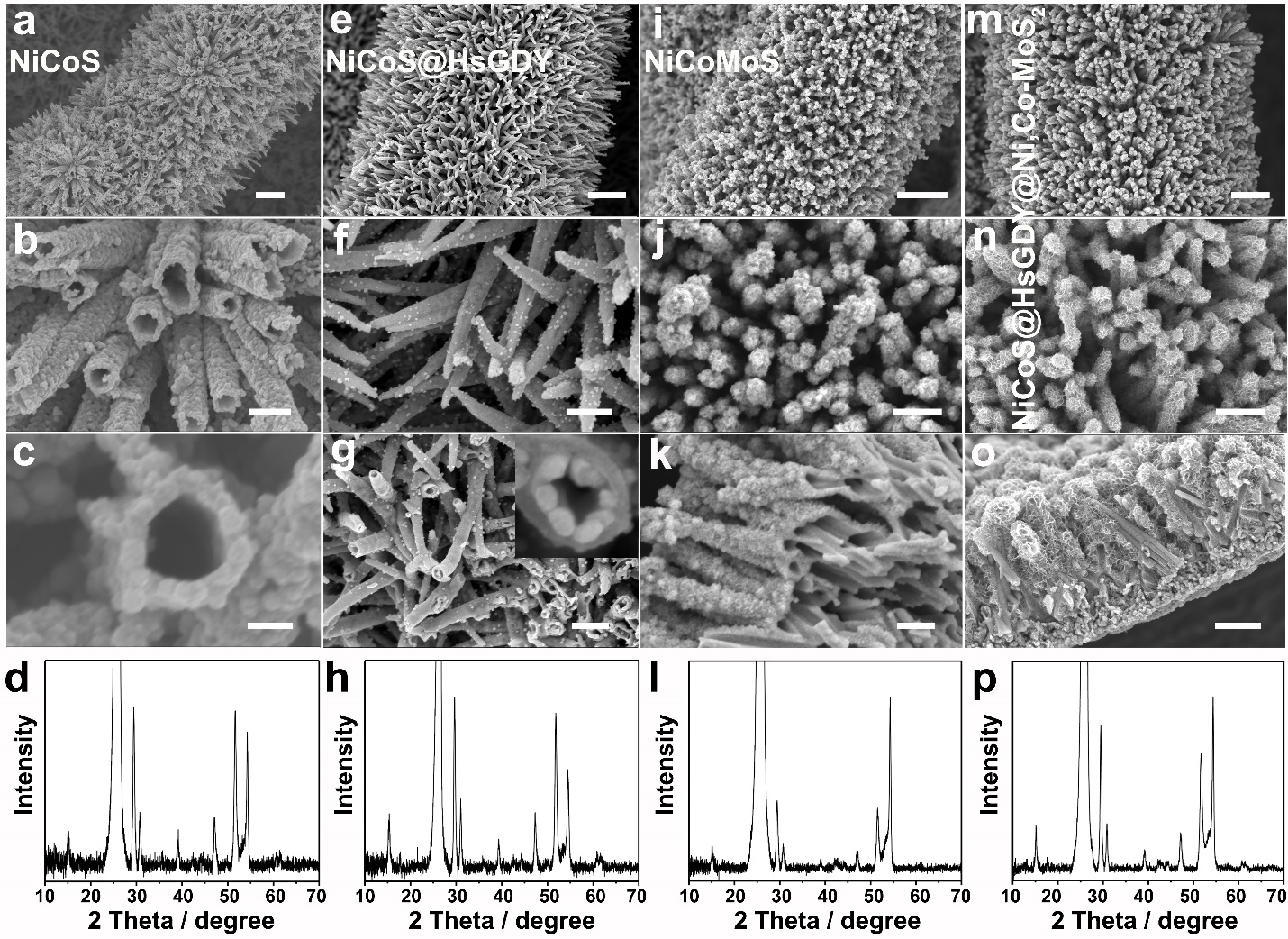


**Supplementary Figure 16** Morphology and structural characterizations of the samples. **a-d** SEM images and XRD patterns of single-layered NiCoS nanotube arrays, **e-h** bi-layered NiCoS@HsGDY nanotube arrays, **i-l** single-layered NiCoMoS nanotube arrays, and **m-p** tri-layered NiCoS@HsGDY@Ni,Co-MoS_2_ nanotube arrays. Scale bars: (**a, e, i, m**) 2 μm, (**b**) 200 nm, (**c**) 100 nm, (**f, j, g, o**) 400 nm, (**k**) 300 nm, (**n**) 500 nm


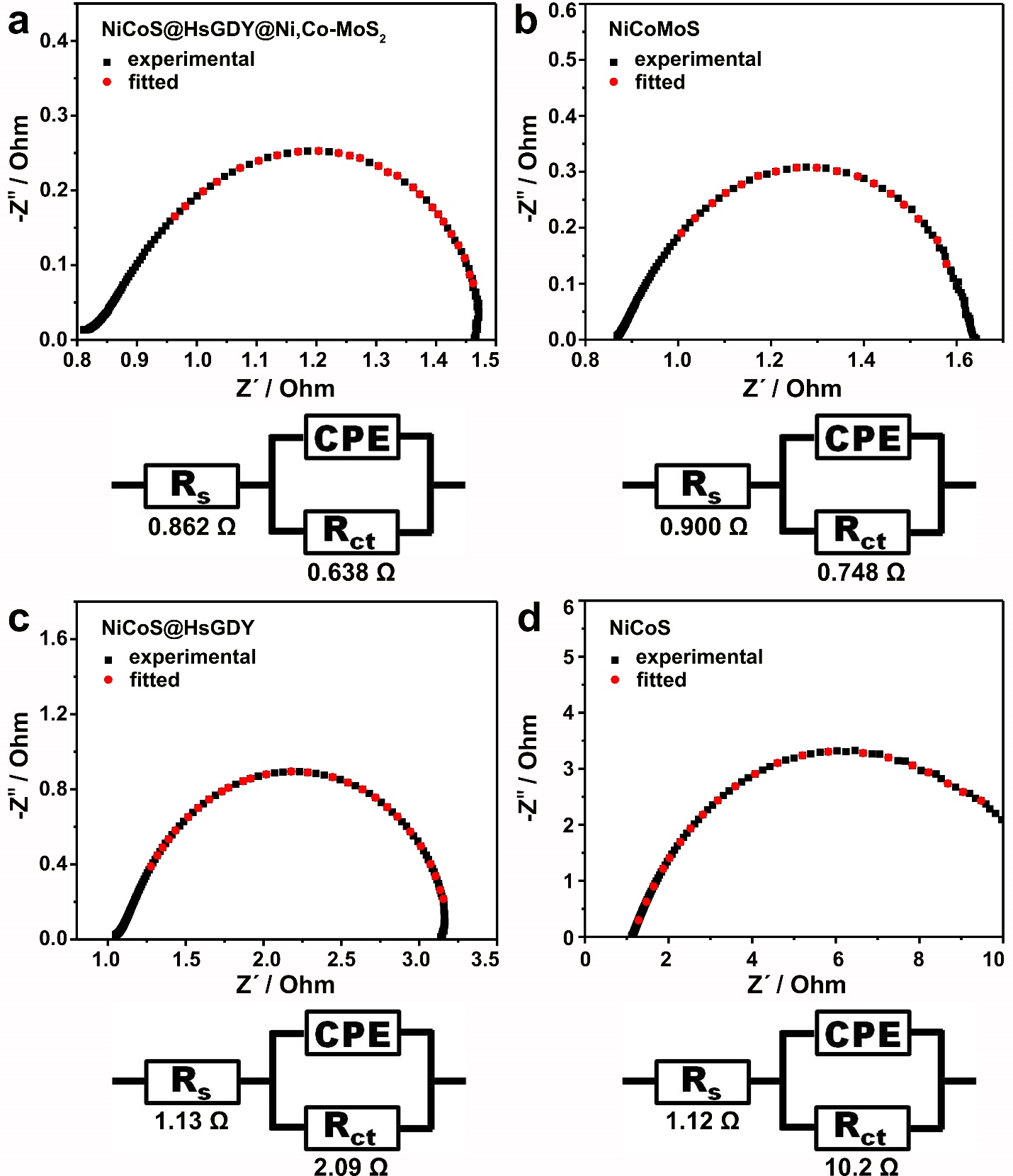


**Supplementary Figure 17** Electrochemical characterizations of the samples. **a** EIS Nyquist plots of NiCoS@HsGDY@Ni,Co-MoS_2_, **b** NiCoMoS, **c** NiCoS@HsGDY, **d** NiCoS and their corresponding equivalent electrical circuit used to model the HER process on these nanoarray electrodes at an overpotential of 200 mV in 0.5 M H_2_SO_4_.

**Supplementary Note 2**

In order to reveal the electron transfer function of the built-in HsGDY layers, the EIS spectra of NiCoS, NiCoS@HsGDY, NiCoMoS and NiCoS@HsGDY@Ni,Co-MoS_2_ are compared. As shown in Supplementary Figure 17, these electrodes all exhibit one capacitive semicircle in the high frequencies, which means that their corresponding equivalent circuits for the HER process are characterized by one time constant and the reaction is kinetically controlled. As a result, the electrical equivalent circuit diagrams with a constant phase element (CPE) are given in their EIS spectra along with fitted curves, which are then utilized to model the solid-liquid interface. As known, the solution resistance R_s_ is independent of overpotential of HER, while the charge transfer resistance R_ct_ determines the electrocatalytic kinetics of HER. The values of R_ct_ decrease significantly from 10.2 Ω to 2.09 Ω when NiCoS is incorporated with HsGDY (NiCoS@HsGDY), suggesting much facilitated electron transfer by introducing HsGDY. A further decrease in R_ct_ value of NiCoS@HsGDY@Ni,Co-MoS_2_ (0.638 Ω) clearly indicates the benefit of the tri-layer structure. When compared with NiCoMoS (0.748 Ω), the lower R_ct_ value of NiCoS@HsGDY@Ni,Co-MoS_2_ (0.638 Ω) also reveals the role of HsGDY as a built-in electron conductive channel during the HER process.


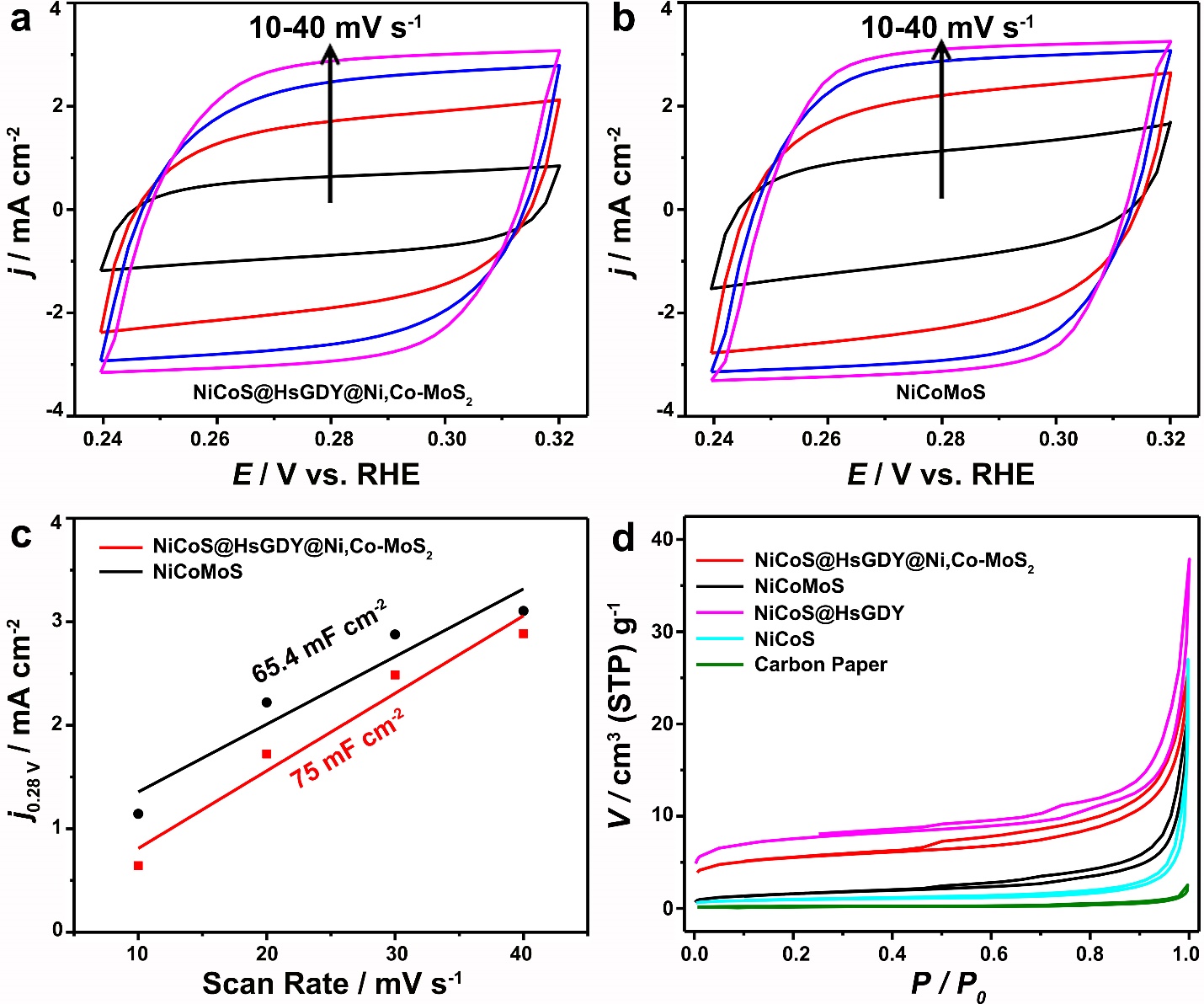


**Supplementary Figure 18** Electrochemical characterizations of the samples. Cyclic voltammograms within the range of no faradaic reactions of tri-layered NiCoS@HsGDY@Ni,Co-MoS_2_ (**a**) and mixed NiCoMoS nanoarrays (**b**), and (**c**) the corresponding variation of double-layer charging current at 0.28 V as a function of scan rate; (**d**) N_2_ adsorption/desorption isotherms of nanoarray electrodes of NiCoS@HsGDY@Ni,Co-MoS_2_, NiCoMoS, NiCoS@HsGDY, NiCoS and carbon paper.


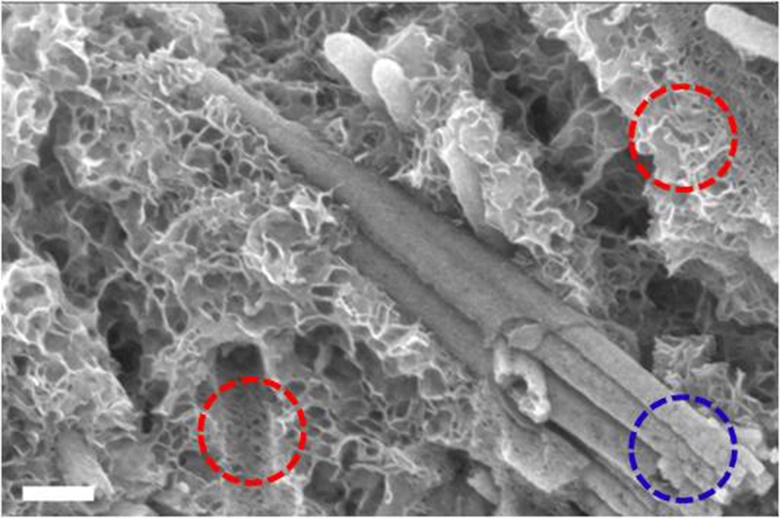


**Supplementary Figure 19** Scanning electron microscopy micrographs. SEM image of hierarchical tri-layered NiCoS@HsGDY@Ni,Co-MoS_2_ nanoarrays taken from some broken areas. Scale bars: 200 nm.


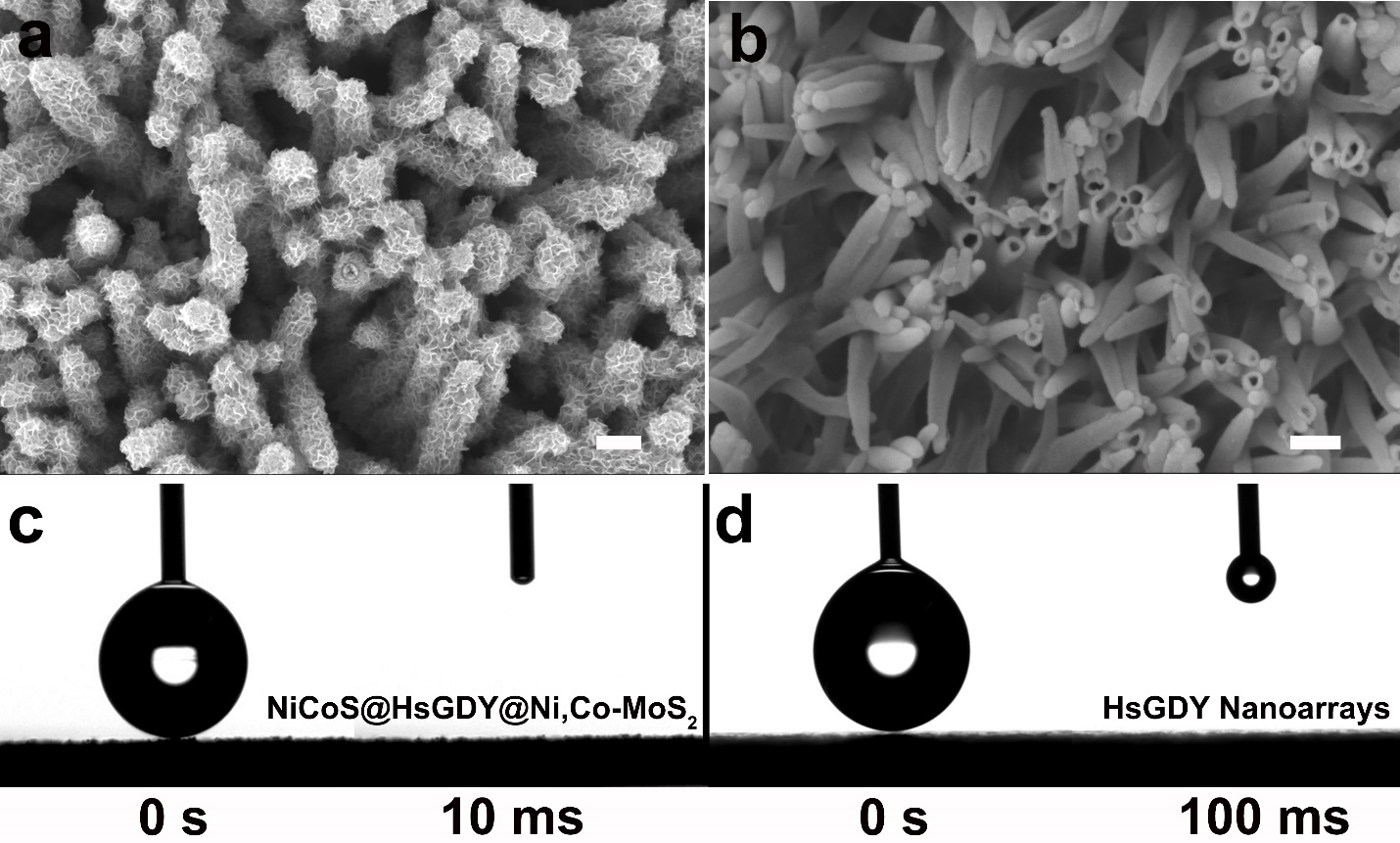


**Supplementary Figure 20** Morphology and structural characterizations of the samples. SEM images and corresponding contact angles of hierarchical tri-layered NiCoS@HsGDY@Ni,Co-MoS_2_ nanoarrays (**a, c**) and HsGDY nanoarrays (**b, d**).

**
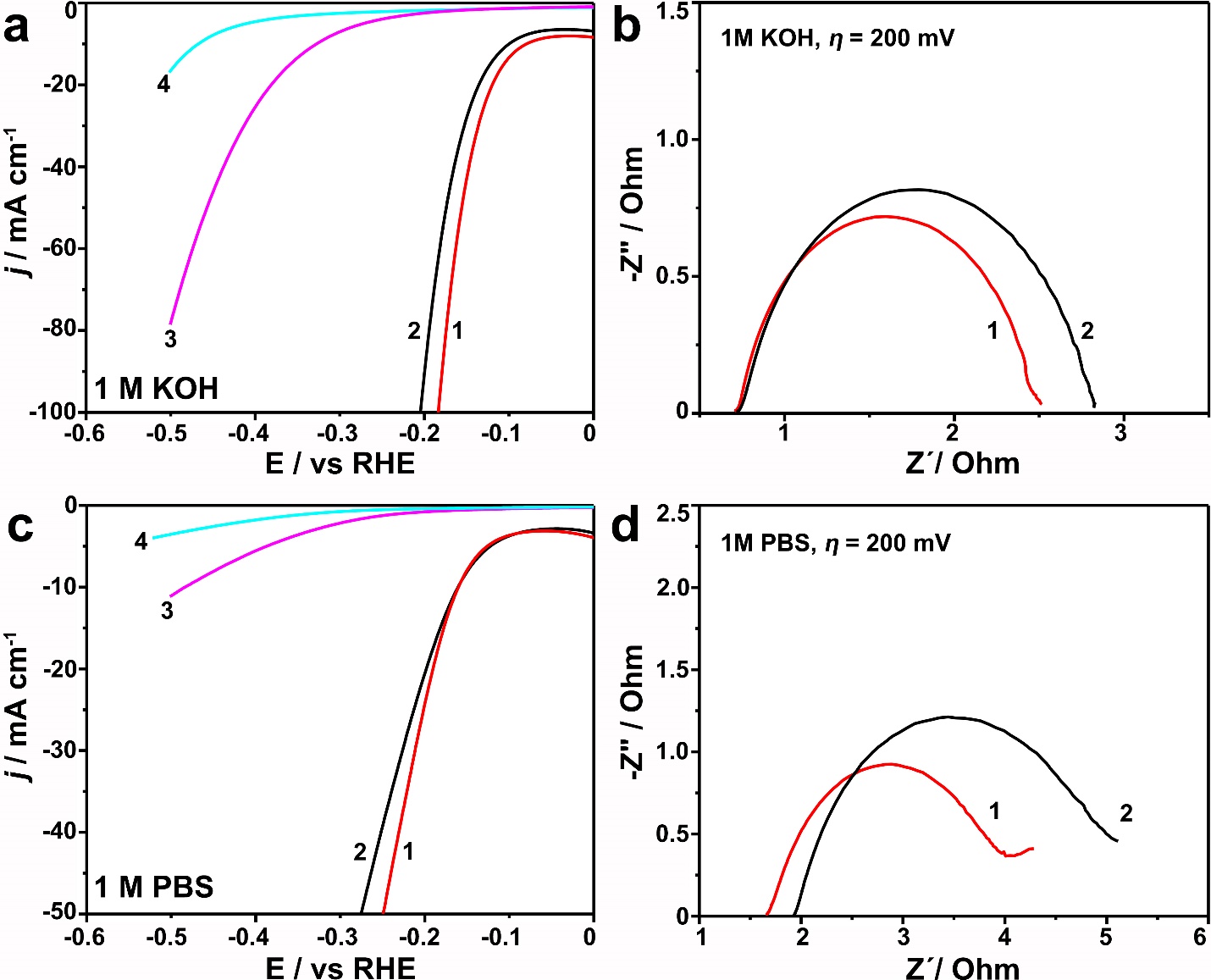
**

**Supplementary Figure 21** Electrochemical evaluations of the samples. **a, c** HER polarization curves with *iR*-compensations and **b, d** EIS Nyquist plots of NiCoS@HsGDY@Ni,Co-MoS_2_ (**1**), NiCoMoS (**2**), HsGDY (**3**) and Carbon paper (**4**) in 1M KOH (**a, b** pH=14.1) and 1M PBS (**c, d** pH=7.1), respectively.


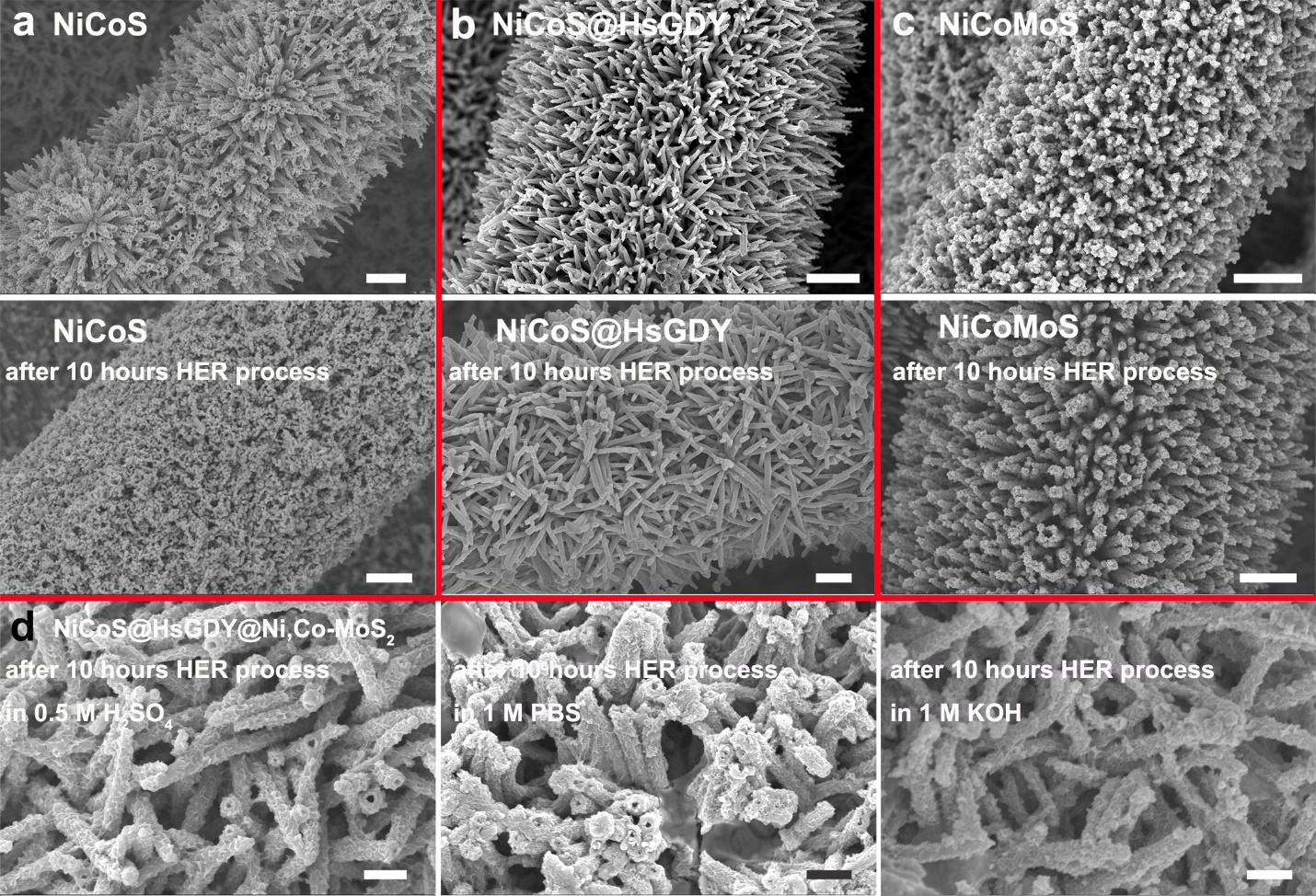


**Supplementary Figure 22** Scanning electron microscopy micrographs. SEM images of NiCoS (**a**), NiCoS@HsGDY (**b**), NiCoMoS (**c**) and their corresponding images after 10 hours HER process performed in 0.5 M H_2_SO_4_; (**d**) SEM images of NiCoS@HsGDY@Ni,Co-MoS_2_ after 10 hours HER process performed in 0.5 M H_2_SO_4_, 1 M PBS and 1 M KOH, respectively. Scale bars: (**a**) 2 μm (top), 1 μm (down), (**b**) 2 μm (top), 1 μm (down), (**c**) 2 μm, (**d**) 400 nm.


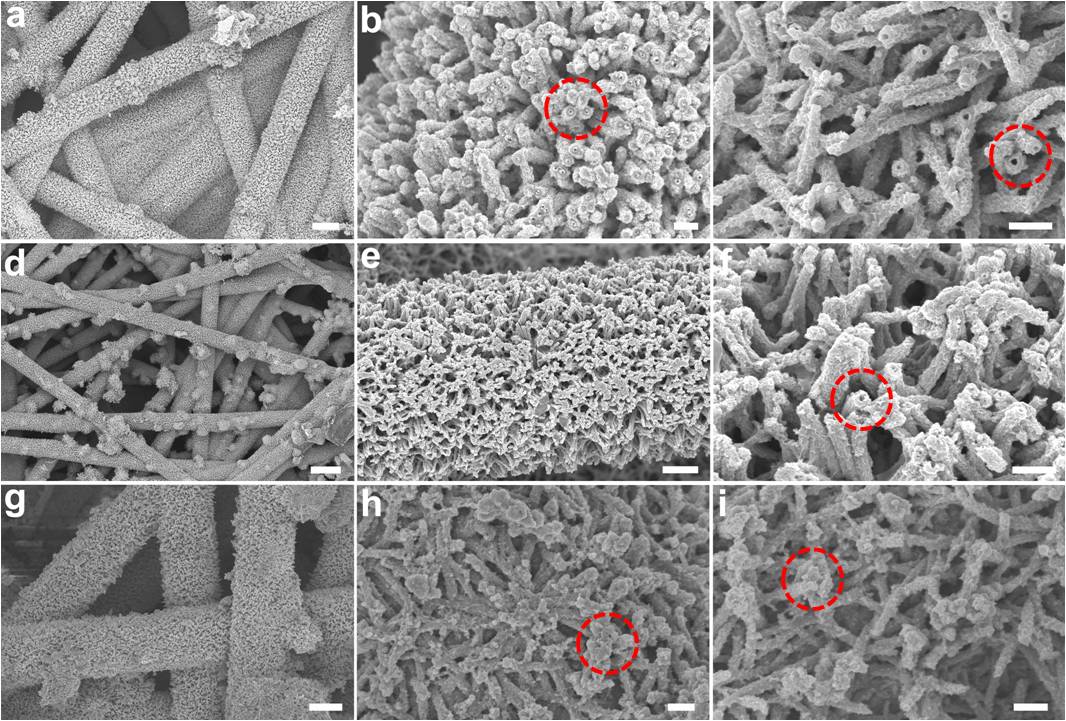


**Supplementary Figure 23** Scanning electron microscopy micrographs. SEM images of the tri-layered NiCoS@HsGDY@Ni,Co-MoS_2_ nanoarrays after 10 hours HER process performed in 0.5 H_2_SO_4_ (**a-c**), 1 M PBS (**d-f**) and 1 M KOH (**g-i**), respectively. Scale bars: (**a**) 10 μm, (**b, c, f, h, i**) 500 nm, (**d**) 20 μm, (**e**) 2 μm, (**g**) 5 μm.

**Supplementary Note 3**

To reveal the property reduction affected by the pH values, we have carefully analyzed the SEM images of NiCoS@HsGDY@Ni,Co-MoS_2_ after 10 hours HER process in different pH values. As shown in Supplementary Fig. 23 a-c, the well-aligned NiCoS@HsGDY@Ni,Co-MoS_2_ nanoarrays with hierarchical tri-layered structures are well-maintained after 10 hours HER process in 0.5 M H_2_SO_4_. These results demonstrate the impressive stability of these tri-layered nanoarrays in acidic electrolyte. With 1 M PBS electrolyte, as shown in Supplementary Fig. 23 d-f, the well-aligned nanoarrays with hierarchical tri-layered are well-maintained when performed in 1 M PBS for 10 hours. These results suggest that the core structure of Ni_3_S_2_/Co_9_S_8_ is stable in 1M PBS electrolyte. The 20% reduction in property after 10 hours HER process in PBS could be due to the partial collapse of the original fluffy Ni,Co-MoS_2_ nanosheets. However, when in 1 M KOH (pH=14.1), although the middle HsGDY layers could effectively protect the core of Ni_3_S_2_/Co_9_S_8_, the partial dissolution/aggregation of the core structures still occurred. As shown in the red circle of Supplementary Fig. 23g-i, although the well-aligned nanoarrays were well reserved at the end of test, some aggregates appeared on the top of some nanowires, which could adversely affect the mass transfer. However, the built-in HsGDY layers lead to the long-term stability of the tri-layered NiCoS@HsGDY@Ni,Co-MoS_2_ nanoarrays beyond the initial stage. As a result, the current decreased 40% in the first 1 hour, but stayed stable in the next 9 hours. In conclusion, the property of NiCoS@HsGDY@i,Co-MoS_2_ is affected by the pH values of the electrolytes, which leads to different current decreases in the first 1 hour. However, the long-term performance is stable, indicating their much improved stability due to the built-in HsGDY layers.

**Supplementary Table 1** Electrochemical evaluations of the samples. Comparison of the electrochemical HER properties of the tri-layered NiCoS@HsGDY@Ni,Co-MoS_2_ nanotube arrays with the reported 2H-MoS_2_, Ni_3_S_2_ and Co_9_S_8_ nanostructures.

| **Electrode**  **Materials** | ***η* _onset_**  **(mV)** | ***η* _10mA cm-2_**  **(mV)** | **Tafel Slope**  **(mV dec^-1^)** | | **Electrolyte** | | **Substrate** | **Ref.** | |
| --- | --- | --- | --- | --- | --- | --- | --- | --- | --- |
| Zn-MoS_2_ | 130 |  | 51 | 0.5 M H_2_SO_4_ | | GC | | | [1] |
| Ni-Mo-S/C | - | 200 | 85.3 | 0.5 M PBS | | Carbon Cloth | | | [2] |
| Ni-Co-MoS_2_ | 125 | 155 | 51 | 0.5 M H_2_SO_4_ | | GC | | | [3] |
| CoMoS_3_ | 75 | 171 | 56.9 | 0.5 M H_2_SO_4_ | | GC | | | [4] |
| NiMo_3_S_4_ | 59 | 257 | 98 | 0.1 M KOH | | GC | | | [5] |
| MoS_2_/Ni_3_S_2_  MoS_2_/NGDY  MoS_2_/Carbon | 50  103 | 110  186  159 | 83  63  56.1 | 1. M KOH   0.5 M H_2_SO_4_  0.5 M H_2_SO_4_ | | Ni Foam  GC  GC | | | [6]  [7]  [8] |
| Cu-NDs/Ni_3_S_2_ |  | 128 | 76.2 | 1.0 M KOH | | Carbon Cloth | | | [9] |
| Ni_3_S_2_/NF |  | 170 |  | 1.0 M PBS | | Ni Foam | | | [10] |
|  |  | 223 |  | 1.0 M KOH | |  |  |  |  |
| Co_9_S_8_@C | 150 |  |  | 1.0 M PBS | |  | | |  |
|  |  | 240 |  | 0.5 M H_2_SO_4_ | | GC | | | [11] |
|  |  | 250 |  | 1.0 M KOH | |  | | |  |
| This work |  | 124  100  160 | 64.3  89.5  98.8 | 0.5 M H_2_SO_4_  1.0 M KOH  1.0 M PBS | | Carbon Paper | | | Our work |

**Supplementary Note 4**

A comparison of the HER performance of the tri-layer nanoarrays in this work with many recently reported nanomaterials with similar compositions has been made. As seen, the HER performance of the trip-layered nanoarrays is better than many of reported materials of 2H-MoS_2_, Co_9_S_8_, Ni_3_S_2_. Moreover, (a) the tri-layered nanoarray is a pH-universal electrode while the 2H-MoS_2_ works only in acid electrolyte.^7,8^ (b) Compared with the Ni_3_S_2_/Co_9_S_8_, the activity and stability of the core structure of Ni_3_S_2_/Co_9_S_8_ in the synthesized tri-layered nanoarrays is considerably promoted thanks to seamlessly coated HsGDY layer. (c) The tri-layered NiCoS@HsGDY@Ni,Co-MoS_2_ shows very favorable HER performance in comparison with other hybrid or doped nanostructures such as MoS_2_/Ni_3_S_2_,^6^ NiMo_3_S_4_,^5^ NiMoS/C,^2^ CoMoS_3_,^4^ Zn-MoS_2_,^1^ Ni-Co-MoS_2_,^3^ Ni_3_S_2_/NF,^10^ Co_9_S_8_/C.^11^

**Supplementary Method 1**

**Materials:** CoCl_2_·6H_2_O, NiCl_2_·6H_2_O, Co(NO_3_)_2_·6H_2_O, urea, CTAB, (NH_4_)_2_MoS_4_, CuI, Pd(PPh_3_)_2_Cl_2,_ N_2_H_4_, KMnO_4_, tetrahydrofuran and trimethylamine were purchased from Sigma-Aldrich. 1,3,5-triethynylbenzene was purchased from Alfa Aesar. NH_4_F was purchased from Acros Organics. All chemicals were used as received without further purification. The carbon fibre paper (*FuelCellsEtc*, AvCarb MGL370, bulk density of 0.46g cm^-3^, thickness of 0.37mm, porosity of 78%, electrical resistivity of 75 mΩcm, area of 2.5×6 cm^2^) was first cleaned by sonication in chloroform, ethanol and deionized (DI) water for 20 min successively, and then heat-treated at 500 ℃ for 1h in air to increase its wettability, And then soaking it in an aqueous solution (150 mL) containing 0.1 M KMnO_4_ for 1 h to form a seed layer.

**Synthesis of Ni(OH)F nanosheet arrays and Co(OH)F nanowire arrays:** Well-aligned Co(OH)F nanowire arrays were fabricated according to a previously reported method.^12^ Briefly, 5 mmol of Co(NO_3_)_2_·6H_2_O, 10 mmol of NH_4_F and 25 mmol of CO(NH_2_)_2_ were dissolved into 35 mL of deionized water under sonication to form a homogeneous solution. Then, the solution was transferred to a 50 mL Teﬂon-lined stainless steel autoclave containing the previously cleaned carbon paper. After heat treatment at 120℃ for 9 h in an electric oven, the autoclave was allowed to cooled down to room temperature, the carbon paper was taken out of the autoclave and cleaned by ultrasonication with DI water and ethanol, to remove the loosely attached products. Ni(OH)F nanosheet arrays were obtained when Co(NO_3_)_2_·6H_2_O was replaced with Ni(NO_3_)_2_·6H_2_O under the otherwise same conditions.

**Synthesis of NiCoS and NiCoS@HsGDY nanoarrays**: In details, NiCoS (Ni_3_S_2_, Co_9_S_8_) and NiCoS@HsGDY nanoarrays were directly transformed from NiCoHC and NiCoHC@HsGDY nanoarrays in the presence of L-cysteine in DMF solution (210 ℃, 15 h), respectively. Then, the carbon paper was taken out of the autoclave and cleaned by ultrasonication with DI water and ethanol to remove the loosely attached products.

**Characterization:** X-ray photoelectron spectroscopy (XPS) studies were carried out in an Axis Ultra instrument (Kratos Analytical) under ultrahigh vacuum (<10^-9^ mbar) by using a monochromatic Al Kα X-ray (hυ = 1486.6 eV) source operated at 150 W. The survey and high-resolution spectra were obtained at fixed analyzer pass energies of 160 eV and 20 eV respectively, and quantified using empirically derived relative sensitivity factors provided by Kratos Analytical. Floating mode was used for samples mounted in so as to avoid differential charging. The C 1s binding energy of adventitious carbon contamination set at 284.8 eV was used as binding energies reference. The data were analyzed with commercially available software, CasaXPS. NMR spectra were acquired using WB Bruker 600 AVANAC III spectrometer equipped with 2.5 mm double resonance MAS Bruker Probe (BrukerBioSpin, Rheinstetten, Germany). The samples were finely ground first and then packed evenly into 2.5 mm zirconia rotor and sealed at the open end with a Vespel cap. The spectra were recorded using cp pulse program from Bruker pulse library with recycle delay time of 5 s. Bruker Topspin 3.2 software (Bruker BioSpin, Rheinstetten, Germany) was used to collect and to analyze the data.

**Supplementary references**

1. Shi Y. *et al*. Energy level engineering of MoS_2_ by transition-metal doping for accelerating hydrogen evolution reaction, *J. Am. Chem. Soc.* **139**, 15479-15485 (2017).
2. Miao J. *et al*. Hierarchical Ni-Mo-S nanosheets on carbon fiber cloth: A flexible electrode for efficient hydrogen generation in neutral electrolyte. *Sci. Adv*. **1**, e1500259 (2015).
3. Yu X.-Y., Feng Y., Jeon Y., Guan B., Lou X. W. & Paik U., Formation of Ni–Co–MoS_2_ nanoboxes with enhanced electrocatalytic activity for hydrogen evolution. *Adv. Mater.* **28**, 9006-9011 (2016)
4. Yu L., Xia B. Y., Wang X. & Lou X. W., General formation of M–MoS_3_ (M = Co, Ni) hollow structures with enhanced electrocatalytic activity for hydrogen evolution. *Adv. Mater.* **28**, 92-97 (2016).
5. Jiang J., Gao M., Sheng W. & Yan, Y., Hollow chevrel-phase NiMo_3_S_4_ for hydrogen evolution in alkaline electrolytes. *Angew. Chem. Int. Ed.* **55**, 15240-15245 (2016).
6. Zhang J. *et al*. Interface engineering of MoS_2_/Ni_3_S_2_ heterostructures for highly enhanced electrochemical overall-water-splitting activity. *Angew. Chem. Int. Ed*. **55**, 6702-6707 (2016).
7. Yu, H. *et al*. Controlled growth of MoS_2_ nanosheets on 2D N-doped graphdiyne nanolayers for highly associated effects on water reduction. *Adv. Funct. Mater*. 1707564 (2018).
8. Yang, L. *et al*. Hierarchical spheres constructed by defect-rich MoS_2_/carbon nanosheets for efficient electrocatalytic hydrogen evolution. *Nano Energy* **22,** 490–498 (2016).
9. Feng J.-X., Wu J.-Q., Tong Y. & Li G.-R. Efficient hydrogen evolution on Cu nanodots-decorated Ni_3_S_2_ nanotubes by optimizing atomic hydrogen adsorption and desorption. *J. Am. Chem. Soc.* **140**, 610-617 (2018).
10. Feng L.-L. *et al*. High-index faceted Ni_3_S_2_ nanosheet arrays as highly active and ultrastable electrocatalysts for water splitting. *J. Am. Chem. Soc.* **137**, 14023-14026 (2015).
11. Feng L. L. *et al*. carbon-armored Co_9_S_8_ nanoparticles as all-pH efficient and durable H_2_-evolving electrocatalysts. *ACS Appl. Mater. Interfaces* **7**, 980-988 (2015).
12. Xiong, D., Li, X., Bai, Z., Li, J., Han, Y. & Li, D. Vertically aligned Co_9_S_8_ nanotube arrays onto graphene papers as high-performance flexible electrodes for supercapacitors *Chem. Eur. J*. **24**, 2339–2343 (2018).
